# Progressive stretch enhances growth and maturation of 3D stem-cell-derived myocardium

**DOI:** 10.7150/thno.54999

**Published:** 2021-04-15

**Authors:** Kun Lu, Thomas Seidel, Xiaochun Cao-Ehlker, Tatjana Dorn, Aarif Mohamed Nazeer Batcha, Christine Maria Schneider, Marie Semmler, Tilmann Volk, Alessandra Moretti, Andreas Dendorfer, Roland Tomasi

**Affiliations:** 1 Walter-Brendel-Centre of Experimental Medicine, University Hospital, Ludwig-Maximilians-University München, Munich, Germany.; 2 Institute of Cellular and Molecular Physiology, Friedrich-Alexander University Erlangen-Nürnberg, Erlangen, Germany.; 3 First Department of Medicine, Cardiology, Klinikum rechts der Isar, Technical University München, Munich, Germany.; 4 Institute for Medical Information Processing Biometry and Epidemiology, Ludwig-Maximilians-University München, Munich, Germany.; 5 DIFUTURE (Data Integration for Future Medicine), Ludwig-Maximilians-University München, Munich, Germany.; 6 DZHK (German Center for Cardiovascular Research), Partner site Munich Heart Alliance, Munich, Germany.; 7 Clinic of Anaesthesiology, University Hospital, Ludwig-Maximilians-University München, Munich, Germany.

**Keywords:** engineered heart tissue, progressive stretch, stem-cell-derived myocardium, maturation, biomechanics

## Abstract

Bio-engineered myocardium has great potential to substitute damaged myocardium and for studies of myocardial physiology and disease, but structural and functional immaturity still implies limitations. Current protocols of engineered heart tissue (EHT) generation fall short of simulating the conditions of postnatal myocardial growth, which are characterized by tissue expansion and increased mechanical load. To investigate whether these two parameters can improve EHT maturation, we developed a new approach for the generation of cardiac tissues based on biomimetic stimulation under application of continuously increasing stretch.

**Methods:** EHTs were generated by assembling cardiomyocytes derived from human induced pluripotent stem cells (hiPSC-CM) at high cell density in a low collagen hydrogel. Maturation and growth of the EHTs were induced in a custom-made biomimetic tissue culture system that provided continuous electrical stimulation and medium agitation along with progressive stretch at four different increments. Tissues were characterized after a three week conditioning period.

**Results:** The highest rate of stretch (S3 = 0.32 mm/day) increased force development by 5.1-fold compared to tissue with a fixed length, reaching contractility of 11.28 mN/mm². Importantly, intensely stretched EHTs developed physiological length-dependencies of active and passive forces (systolic/diastolic ratio = 9.47 ± 0.84), and a positive force-frequency relationship (1.25-fold contractility at 180 min^-1^). Functional markers of stretch-dependent maturation included enhanced and more rapid Ca^2+^ transients, higher amplitude and upstroke velocity of action potentials, and pronounced adrenergic responses. Stretch conditioned hiPSC-CMs displayed structural improvements in cellular volume, linear alignment, and sarcomere length (2.19 ± 0.1 µm), and an overall upregulation of genes that are specifically expressed in adult cardiomyocytes.

**Conclusions:** With the intention to simulate postnatal heart development, we have established techniques of tissue assembly and biomimetic culture that avoid tissue shrinkage and yield muscle fibers with contractility and compliance approaching the properties of adult myocardium. This study demonstrates that cultivation under progressive stretch is a feasible way to induce growth and maturation of stem cell-derived myocardium. The novel tissue-engineering approach fulfills important requirements of disease modelling and therapeutic tissue replacement.

## Introduction

Engineered heart tissues (EHTs) from human induced pluripotent stem cell-derived cardiomyocytes (hiPSC-CMs) have great potential as substrates for drug screening, disease modeling, and cardiac research, but structural and functional immaturity of the tissues still implies limitations that need to be overcome 1,2. The fundamental idea to generate artificial tissues by close replication of natural heart development has guided the establishment of robust protocols of cardiomyocyte differentiation and tissue formation 3,4. These protocols are based on standardization and cost-effectiveness to form highly scalable 3D tissues and not only single-cell models. However, the resulting cellular phenotype is immature, i.e., ultrastructure and functional properties resemble early embryonic rather than adult cardiomyocytes. Therefore, more recent research has focused on the significance of mechanical and electrical cues to advance hiPSC-CMs to a more adult phenotype 2,5-7. In particular, the assembly of hiPSC-CMs to 3-dimensional EHTs has been reported to foster progressive cellular maturation by permitting not only electrical but also hydrodynamic, metabolic and/or mechanical (i.e., biomimetic) stimulation 2,8-11. Despite these advances, a fully mature tissue structure concerning the size and alignment of cardiomyocytes, and physiological tissue mechanics has not yet been achieved 1,2,12-14.

In heart development, the characteristic properties of adult cardiomyocytes arise particularly during the postnatal phase, in which maturation of the myocardium is governed by growth and increasing mechanical load 7,15. Current protocols of EHTs generation, however, cannot properly simulate these conditions. They are mostly based on the differentiation of pluripotent cells into early cardiomyocytes (hiPSC-CMs), which require attachment to externally provided scaffolds for tissue formation. Commonly, hiPSC-CMs are incorporated into natural polymer hydrogels as scaffold materials 16,17, with extracellular matrices mostly consisting of collagen, fibrin or Matrigel 1,2,4. Scaffold protein composition and cell population both play critical roles in the maturation of EHTs, influencing the compaction, tissue stiffness, and active force production 16. To date, EHTs are typically composed of high amounts of extracellular matrix and low cell density to ensure proper initial stiffness, while passive tension is generated by hydrogel compaction 2. Due to these circumstances, the maximum elongation and the size of cardiomyocytes are limited, and the contractile performance of the EHT is frequently impaired by low diastolic compliance 5.

To induce a more physiologic growth, we propose a new approach for the generation of cardiac tissues that is based on elongation and growth of EHTs under the application of defined diastolic load. The new technique separates tissue assembly from maturation. First, the assembly of solid tissue is promoted by high cell density and low amounts of extracellular matrix, supporting cellular cohesion. Second, maturation and growth of the immature myocardium are induced by elaborated biomimetic stimulation 18, which includes successive tissue expansion (progressive stretch). Here, such treatment was provided by a custom-made biomimetic tissue culture system that permits the application of defined preload and mechanical load in conjunction with continuous electrical stimulation, medium agitation, and real-time force measurement 18. These conditions of biomimetic culture had been validated by their ability to maintain viability and mechanical performance of adult human myocardial slices for several months 18, and therefore seemed appropriate for EHT cultivation. In particular, the biomimetic system permitted daily expansion of cardiac tissues by freely adjustable posts, as well as a continuous recording of preload and contractility, which both might be considered as integrative functional markers of myocardial tissue maturation.

This study characterizes the structural and functional consequences of stretch-induced myocardial maturation. It demonstrates the feasibility of the new approach and shows that progressive stretch enhances EHT maturation greatly in terms of contractility, tissue compliance, size and alignment of cardiomyocytes, electrophysiology, and excitation-contraction coupling. We suggest that selective optimization of assembly and subsequent biomimetic maturation of EHTs will help to fulfill the potential of hiPSC-CMs.

## Materials & methods

### Cell culture and differentiation of hiPSC-CMs

**Figure 1:**
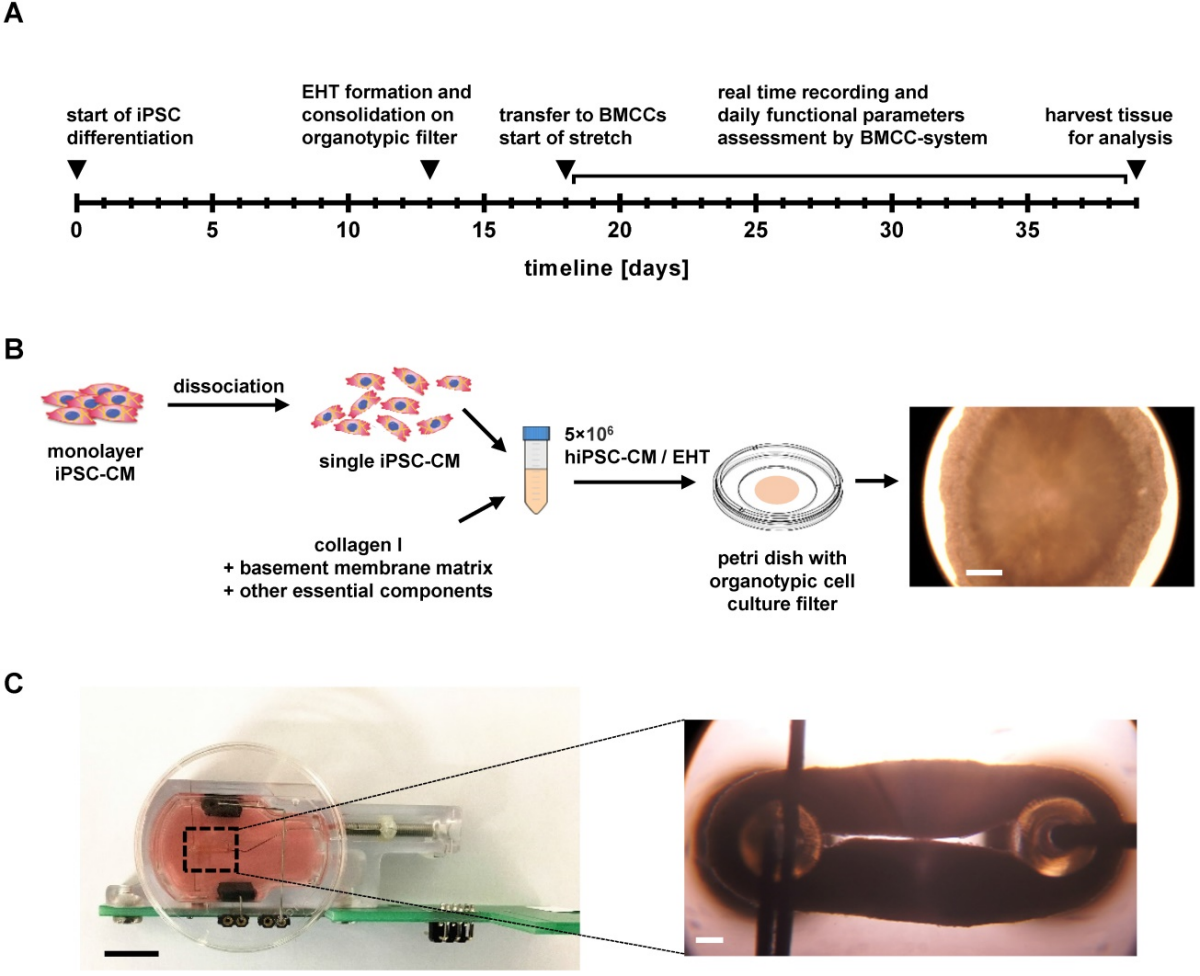
** Experimental design and EHT formation.** (A) Workflow of the experimental design. (B) Schematic representation of EHT formation and detailed presentation of a discoid primary EHT. Scale bar: 0.5 mm. (C) Detailed view of EHT fixation in the biomimetic culture chambers (BMCCs). Scale bar left: 10 mm; scale bar right: 0.5 mm.

Pluripotent stem cells were generated by reprogramming skin fibroblasts (ID MRIi004-A) of a healthy human donor according to established methods 19. The study participant provided written informed consent and the investigation conformed to the principles outlined in the Declaration of Helsinki. For maintenance culture, hiPSCs were expanded in matrix-coated dishes (Geltrex LDEV-Free, Gibco), using Essential 8 medium (Gibco), which was changed on a daily basis. Cells were split at 85% confluency, using EDTA for dissociation (Versene solution; Gibco). A state-of-the-art differentiation protocol for hiPSC-CMs using small molecules to induce differentiation was adapted from Chen and colleagues 20. Briefly, cardiac differentiation was initiated at about 85% confluence by applying chemically defined factors for 24 h (medium A) and subsequent 48 h (medium B, PSC Cardiomyocyte Differentiation Kit; Gibco). After 72 hours, differentiation of hiPSC-CMs was continued for 12 days with Cardiomyocyte Maintenance Medium (PSC Cardiomyocyte Differentiation Kit; Gibco) that was changed every other day. The workflow is displayed in Figure 1A.

### Primary tissue assembly

Differentiated hiPSC-CMs were dissociated with TrypLE Select Enzyme (Gibco) for 8 min, followed by collagenase II (1.5 mg/mL, Sigma-Aldrich) for 7 min. Cells were dispersed in EB6 medium (Supplemental Table S1) and centrifuged at 390 × g for 5 min. The cell pellet was suspended in EB6 medium to which 0.55 mg/mL bovine collagen I (Gibco), 0.08 mg/mL Geltrex (LDEV-Free, Gibco), and 1% RevitaCell supplement was added (modified from 21), reaching a cell concentration of 1.1 × 10^5^ cells/µL. Next, 55 µL of the cell-matrix mixture was pipetted on a 30 mm organotypic filter (PICMORG50, Merck Millipore), forming a disc of approximately 8 mm diameter and 2 mm thickness. After solidification of the tissue disc (30 min at 37 °C), 1 mL of EB 6 medium was added below the filter and was exchanged every other day for a total of 5 days in culture (Figure 1B; Figure S1A).

### Biomimetic culture

**Figure 2:**
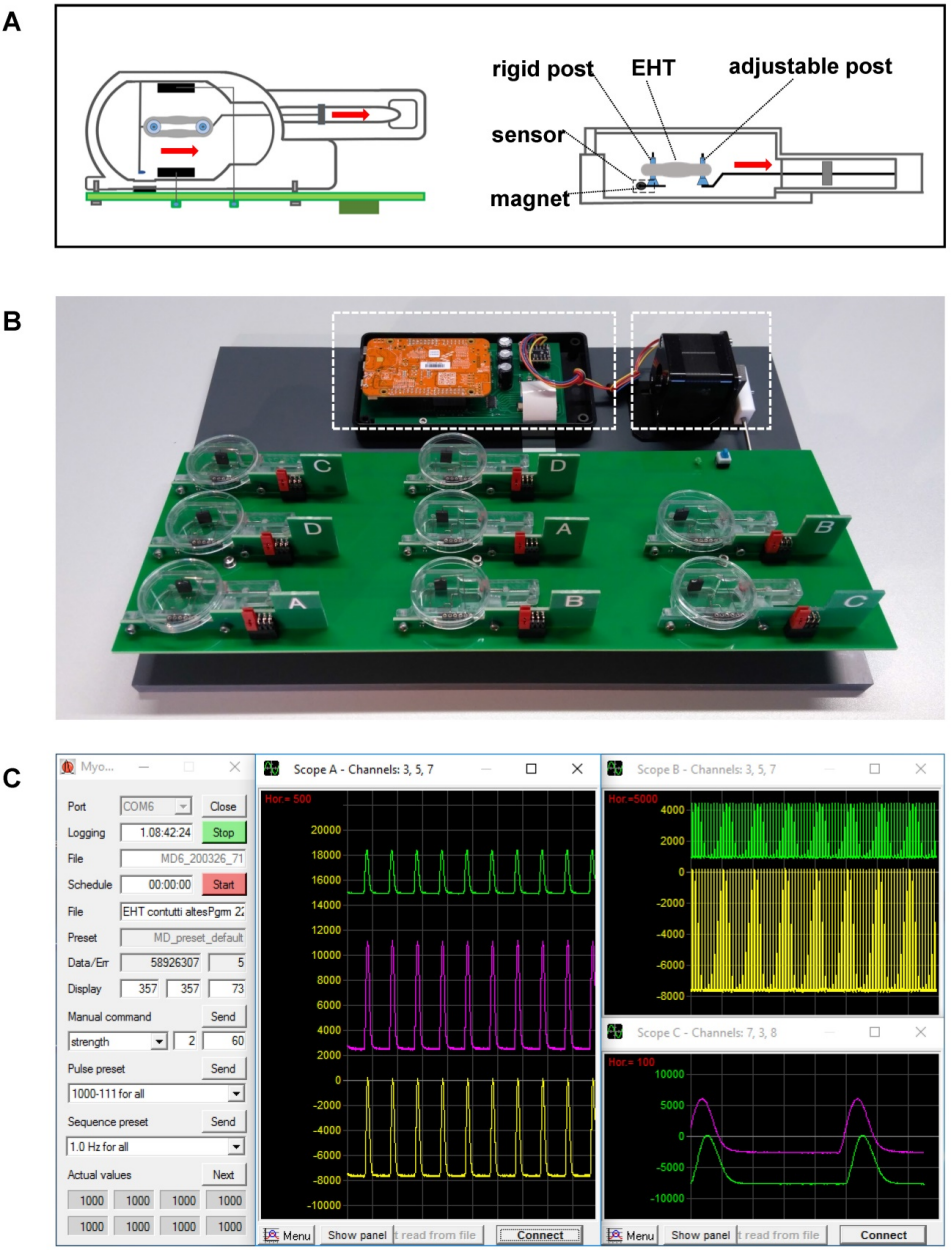
** A novel bioreactor enables stretch conditioning under enhanced biomimetic conditions.** (A) Schematic illustration of mechanical fixation of the primary EHT in a biomimetic culture chamber (BMCC). Red arrows indicate the stretching direction. (B) Setup of the BMCC system. A micro-controller (left dashed box) collects contraction data, generates stimulation pulses, and controls rocking of the BMCC platform by a stepper motor (right dashed box). (C) Computer interface of BMCC system. Data storage and stimulation parameters are set on a control panel (left). Tissue forces are displayed at arbitrary time scales in freely assignable oscilloscope windows (middle and right panels). Each peak represents one contraction.

The system for biomimetic cultivation has been developed by our group for the long-term maintenance of adult human myocardial slices 18. Since all biomimetic features of the cultivation device, such as defined preload, elastic systolic load, continuous bipolar stimulation, and medium agitation, have been found to be essential for the stable performance of cultured human myocardium 18, we utilized the established parameters as baseline conditions for EHT maturation. The biomimetic cultivation chambers (BMCCs) provide elastic mounting, length adjustment, electrical stimulation, and continuous force measurement by magnetic position detection, as described by Fischer et al. 18. Modified chambers were built from injection molded polystyrene blanks (Proto Labs, Feldkirchen, Germany) and utilized a 0.16 mm diameter spring wire (stainless steel, grade 1.4401) for generating an elastic compliance of 71 mm/N (Figure 1C; Figure 2A). Forces developed by the tissues were calculated according to the spring constant and the displacement of a small magnet at the free end of the spring wire, whose magnetic field was detected by an integrated 3D-sensor (FXOS8700, NXP Semiconductors, USA). The posts for EHT mounting were covered with polyethylene tubes (0.96 mm outer diameter, Portex, Smiths Medical, Dublin, USA) that had a flattened bottom end for stable support of the tissues. EHTs were stimulated by bipolar, current-controlled electrical pulses (2 × 1 ms duration, 50 mA, 1 Hz), which were applied by graphite electrodes (6 × 8 mm^2^, CG1290, CGC Klein, Germany) placed at either side of the EHTs (Figure 2A). Eight BMCCs were assembled on a rocking platform driven by a gearless stepper motor and were operated by a dedicated microcontroller (NXP K22F) for autonomous stimulation and continuous force acquisition (Figure 2B). The integrated rocker minimized heat dissipation within the CO_2_-incubator and promoted oxygen uptake by tilting the BMCCs around their longitudinal axis at 12° angle and 60 rpm. With this technique, we achieved pO_2_ levels between 70-100 mmHg in the medium. The culture system was operated within a standard incubator (37 °C, 3% CO_2_, 80% humidity), and was connected via USB to an external computer executing a custom-made software for data acquisition and stimulation control (Figure 2C). Forces developed by the tissues were recorded at 2 ms intervals and were imported into LabChart Reader software (ADInstruments, Australia) for further analysis.

### Stretch conditioning

Primary myocardial tissue discs were transferred from the organotypic filters to the BMCCs containing 2.4 mL pre-warmed (37 °C) modified EHT culture medium (adapted from 22, Table S1, Figure S1). The discs were mounted in the BMCCs by puncturing their centers with adjacently positioned holding posts, which subsequently were adjusted and placed at 3 mm distance. In that way, a ring-shaped EHT was formed that immediately was exposed to electrical stimulation (1 Hz and 50 mA current) and stretch conditioning. EHTs were randomly assigned to one of four groups (S0-S3) and distended once a day by manually moving the sliding fixation hook (adjustable post) at four different increments (S0 = 0 mm/day, S1 = 0.08 mm/day, S2 = 0.16 mm/day, S3 = 0.32 mm/day). The medium was partially (1.6 mL) exchanged every other day with the addition of 0.1 nmol/L 3,3′,5-triiodo-L-thyronine (Sigma-Aldrich). EHTs were cultured for a period of 21 days in 20 independent experiments. Tissue maturation was characterized by continuous force measurements during the cultivation phase, and with endpoint analysis after 21 days.

### Biomechanical analyses

Systolic and diastolic forces of the EHTs were continuously assessed throughout the whole cultivation period. Changes in twitch force, diastolic force, and stimulation threshold were determined. After 3 weeks of culture, EHTs were transferred to an organ bath, and various biomechanical parameters were assessed, as previously described 23, (Methods Supplement). Contractility and elastic modulus were related to the alpha-actinin ^+^ cross-section area of the EHTs.

### Function and gene expression of stretch-conditioned EHTs

After 21 days of exposure to various stretch conditions, EHTs were removed from BMCCs and were analyzed for beta-adrenergic response, Ca^2+^-handling, electrophysiology, gene expression, and tissue structure. Transients of intracellular Ca^2+^ were determined by fluorescence imaging after Fluo-4 loading. Membrane potential was recorded with intracellular electrodes under field stimulation 18. Expression of genes relevant for excitation/contraction coupling, sarcomere structure, or indicating an adult phenotype of cardiomyocytes 22 was analyzed by standard real-time PCR assays. A detailed description of these analyses is given in the Methods Supplement.

### Quantitative morphology of stretch-conditioned EHTs

EHTs were fixed under maintained stretch with 4% paraformaldehyde for one hour. For immunostaining, tissues were equilibrated with 30% sucrose and cryo-sectioned to 20 µm thickness. After permeabilization (1% Triton X-100, 1h), staining was performed with primary antibody to sarcomeric actinin (monoclonal anti-alpha-actinin sarcomeric antibody, 1:100, Sigma-Aldrich), appropriate secondary antibody (goat anti-mouse IgG (H+L) highly cross-adsorbed, Alexa Fluor 546, 1:100, Invitrogen), fluorescent wheat germ agglutinin (WGA conjugated to CF633 at 40 µg/mL, Biotium CF), and DAPI (Invitrogen). In the case of mitochondria visualization, staining was performed with primary antibody to the subunit of the cytochrome c oxidase (rabbit polyclonal MT-CO2 antibody, 1:50, Invitrogen) and appropriate secondary antibody (Alexa Fluor 488 anti-Rabbit IgG 1:500, Invitrogen A-21441). An exact staining protocol is given in the Methods Supplement. The immunostained tissues were visualized using a confocal microscope (inverted Leica SP8X WLL). Using the cell counter plugin of ImageJ, the sarcomere length was measured along the long axis of cells displaying clear striations. A minimum of five measurements per cell were obtained from eleven replicates. The cardiomyocyte cross-section was measured in a minimum of 50 actinin-positive cells of each sample. Morphometric measurements were performed in a blinded fashion. Myofibril alignment was imaged with the second harmonic generation technique according to a previously published method 24.

### Cell morphology and alignment

Paraformaldehyde-fixed EHTs were stained with anti-alpha actinin, WGA-AF647 conjugate (ThermoFisher) and DAPI, and were embedded in Fluoromount on a standard microscope slide. Imaging was performed with a Zeiss LSM780 confocal microscope, using a 63 × oil immersion lens and a pixel size of 0.1 × 0.1 µm^2^. Two-dimensional scans covering an area of 1-3 mm^2^ were acquired with a tile overlap of 10% and subsequent automated stitching (Zeiss ZEN Imaging Software). Images were noise-filtered and deconvolved with measured point spread functions 25. Using histogram-based thresholds and morphological closing operators, myocyte nuclei were segmented from the DAPI signal. Subsequently, nuclei were used as seeds to run a morphological watershed segmentation with the WGA intensity as a gradient image. The resulting segments outlined cells present in the tissue (Figure S3). The alpha-actinin signal was segmented with a histogram-based threshold (mode + 1 SD) 26. To restrict analysis to cardiomyocytes with their centers inside the optical section, only segments with an alpha-actinin area fraction ≥ 0.07 and a nucleus size ≥ 5 µm^2^ were used for morphological analysis. Morphological analysis was performed with custom-written Matlab scripts (version 2019a) and comprised calculation of cell area, cell length and main axes orientation (principal component analysis). The standard deviation of the main axis orientation of each cell and its first- and second-degree neighbors (dispersion) was used as a measure of local cell alignment.

### RNA sequencing and bioinformatics analysis

RNA was prepared using Trizol (Life Technologies) following the manufacturer's instruction. RNA integrity was assessed with the Agilent Bioanalyzer 2100. Total RNA was subjected to library preparation (SENSE Total RNA-Seq Library Prep Kit, Lexogen) and RNA-sequencing on an Illumina HighSeq-2000 platform (SR 50 bp; > 25 Mio reads/sample). Sequence images were transformed with the Illumina software BaseCaller to bcl files, which were demultiplexed to fastq files with CASAVA (v1.8.2). The read counts of genes and transcripts were measured by Salmon (v0.14.1). The counts were normalized (TMM) using edgeR (v3.28.1) and the differential expression was performed using limma (v3.42.2, voom with quality weights). Gene ontology (GO) analysis was performed for those differentially expressed genes (adj p-value < 0.1) using topGO (v2.38.1).

### Quantitation and statistical analysis

Data are shown as mean +/- standard error of the mean (SEM). One-way or two-way ANOVA was used to verify treatment effects, as appropriate. The post-hoc pairwise analysis was done using Tukey's Honest Significant Difference test or Dunnett's multiple comparison test. All statistical analyses were performed with GraphPad Prism 7. Statistical significance was accepted at an error level of p < 0.05. The number of independent experiments performed for each data set is detailed in the figure legends.

### Data availability

Source data are available for all quantitative data shown in figure panels of the article and can be obtained from the corresponding authors. The study used a combination of commercial and open-source software packages, which are specified in the methods section. Computer software developed for stimulation control and data recording, as well as technical details of culture chambers and electronic devices are available from the corresponding authors upon reasonable request. There are no restrictions to data availability.

## Results

### Primary organoids of hiPSC-CMs undergo force-directed remodeling during progressive stretch

The standard protocols for stem cell culture and cardiomyocyte differentiation yielded a monolayer network that displayed first contractile activity at day 8 of differentiation (Figure S2A). Flow cytometric analysis revealed that this cell population consisted of 69 ± 1% of cells being double positive for cardiac troponin (cTNT) as well as alpha-actinin, which were therefore regarded as hiPSC-CMs (Figure S2B).

A feasible approach of EHT formation was introduced with a two-step process of cell assembly and biomimetic training (Figure 1A), as the initial attempts to form EHTs with established procedures failed because of high stiffness or late compaction of the primary tissues. For the most rapid formation of mechanically stable EHTs, the adherent cells were dissociated, mixed with collagen-based matrix, and were assembled on the surface of an organotypic filter (Figure 1B, Figure S1B). This approach promoted cellular contacts by rapid removal of excess medium, resulting in the formation of a flat cardiac microtissue that commenced synchronous spontaneous contraction within 24 hours. During the next five days, the tissue continued to condense, and compacted from 8 mm diameter to 5 mm thus attaining sufficient mechanical stability for the transfer to biomimetic chambers and subsequent stretch application. Mechanical fixation to the holding posts of BMCCs was achieved by puncturing each disc at the center and expanding the gap to 3 mm distance (Figure S1C, Figure 2A). This mounting procedure reliably produced ring-shaped EHTs whose immediate development of contractile force indicated restriction of mechanical damage to the puncture site. During three subsequent weeks of stretch conditioning, the cardiomyocytes assembled along with force trajectories between the posts, forming elongated rings of cardiac muscle whose dimensions were largely determined by the external extension (Figure 1C, Figure S1). No tearing of EHTs occurred at low rates of distension (S0-S2 groups), but 7 out of 20 EHTs ruptured spontaneously during stretch application in the group of most intense stretch (S3). In some cases, the elongated sides of an EHT came in close contact and fused. This did not appear to affect EHT performance and was not affected by the stretch application.

### Mechanical, electrophysiological and metabolic properties of EHTs change during the conditioning period

**Figure 3:**
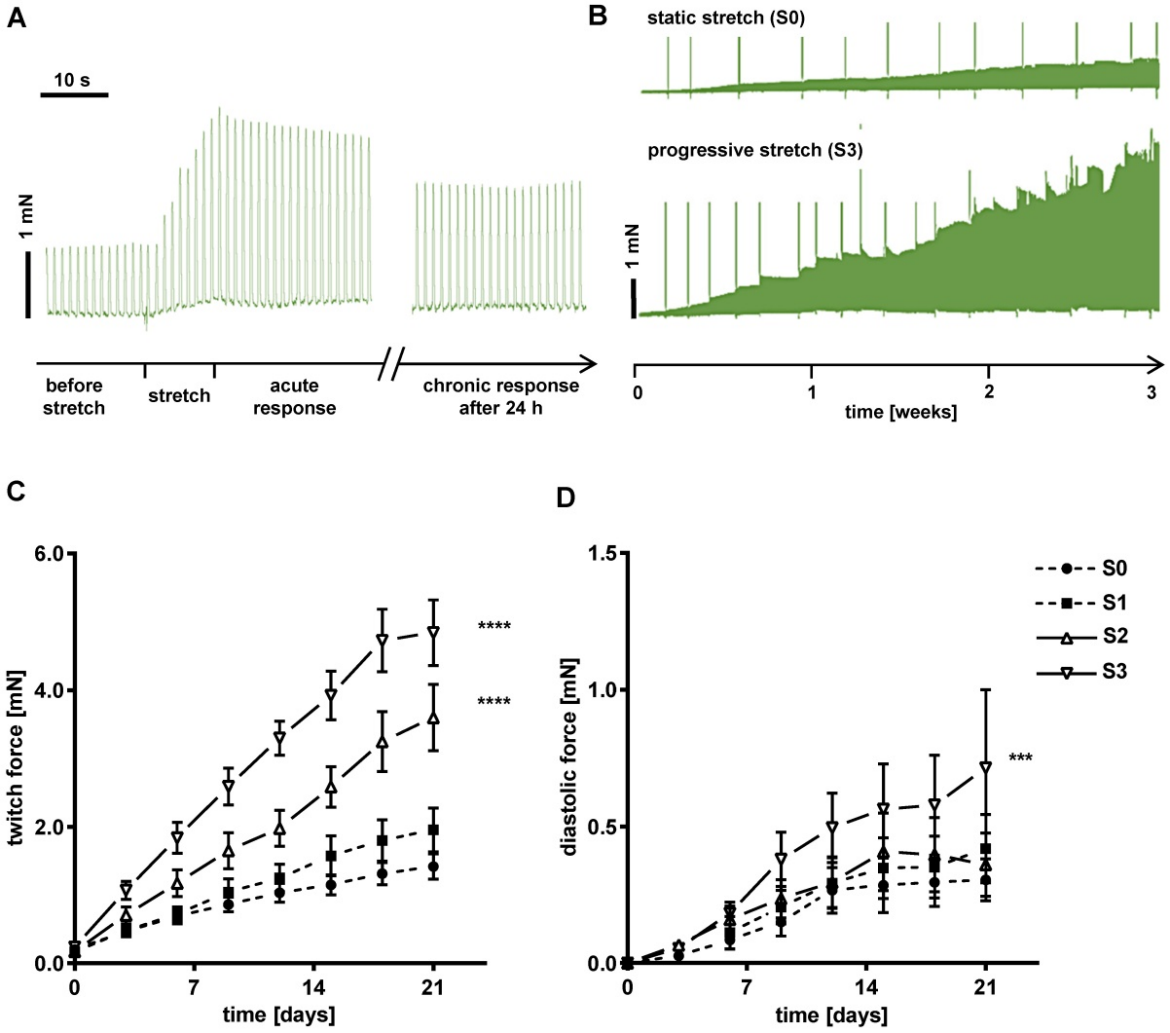
** EHTs gain in contractility and preload during stretch conditioning.** (A) Representative real-time recording of a single stretch manipulation, each peak represents one contraction. (B) Representative twitch force recording of static (S0) and progressive stretch (S3) conditions over a period of three weeks. Periodic breakdowns of contraction force correspond to medium exchange intervals (36-48 h). (C) Twitch force development in groups exposed to different intensities of stretch. (D) Diastolic force development in groups exposed to different intensities of stretch. (C) and (D): static (S0), low (S1), moderate (S2) and high stretch (S3); n = 10 EHTs per group. Two Way ANOVA, Tukey's multiple comparison test vs. static stretch, ***p < 0.001, ****p < 0.0001.

**Figure 4:**
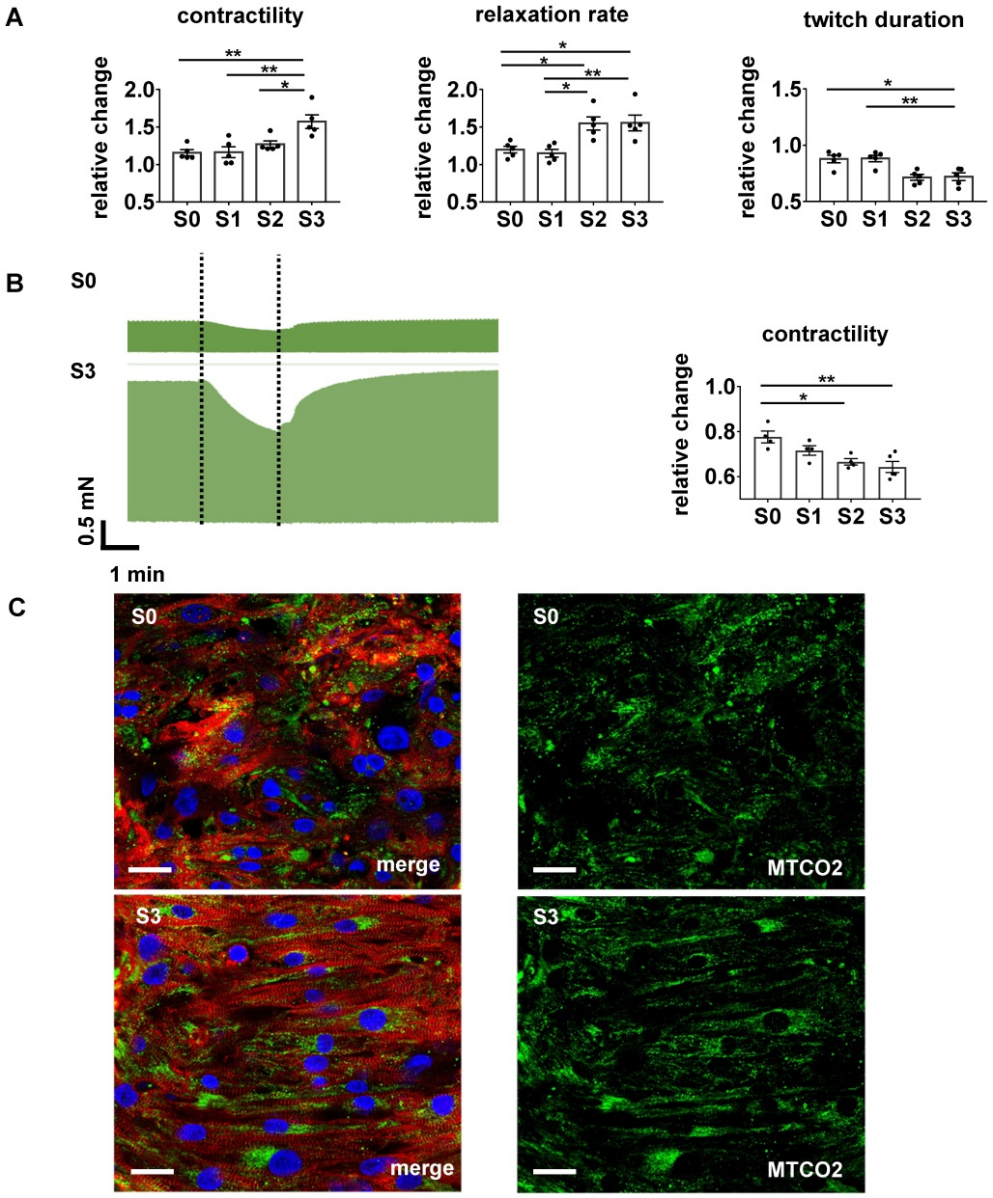
** Stretch conditioning improves adrenergic responsiveness and O_2_ consumption.** (A) Effects of beta-adrenergic stimulation. From left to right: responses to isoprenaline (1 µmol/L) of twitch force, relative relaxation rate, and contraction duration normalized to pre-treatment values (S0-S3, n = 5). One Way ANOVA, Tukey's multiple comparison test, *p < 0.05, **p < 0.01. (B) Contractility response to hypoxia condition. Left: Representative recording of twitch force during hypoxia simulated by 2 min cessation of medium agitation in EHTs conditioned at S0 or S3 stretch. Right: Relative decrease of twitch force induced by 2 min simulated hypoxia in EHTs conditioned under various rates of stretch (S0-S3, n = 5). One Way ANOVA, Kruskal-Wallis test vs. static stretch, *p < 0.05. **p < 0.01. (C) Immunofluorescent images of EHTs cultured under S0 or S3 conditions. Left pictures: Longitudinal sections stained for alpha-actinin (red), subunit of the cytochrome c oxidase in mitochondria - MTCO2 (green) and DNA (blue). Right pictures: MTCO2 component of left pictures. Scale bar: 25 µm.

Force developed by each tissue was continuously recorded during three weeks of stretch conditioning with a sampling rate of 500 Hz (Figure 2C). EHTs at constant length (S0, static stretch) gained contractility over the three weeks' treatment period and developed diastolic force that reached a constant level after about 12 days (Figure 3C-D). In stretched tissues, each step of additional distension induced an acute increase in twitch force (Figure 3A). Despite some subsequent decline of twitch force, the enhancement partially persisted for 24 h and accumulated over the three weeks of progressive stretch (Figure 3B). Any systematic distension enhanced the amplitude of contraction, but only the highest rate of stretch (S3) produced an accumulation of diastolic preload (Figure 3C-D). During the first days of culture, EHTs displayed rapid spontaneous activity, which interfered with the external stimulation. The rate of spontaneous activity fell to less than 1 Hz, so that a consistent 1:1 response to the 1 Hz stimulation was achieved within a week. As a further indicator of electrical maturation, EHTs developed an all-or-nothing force response to electrical stimulation with a progressive reduction of stimulation threshold (Figure S4). The described phenomena of electrical maturation were not significantly related to the applied stretch. O_2_ consumption of the tissues was estimated by the decline in contractility during 2 min cessation of medium agitation. The rate of relative decline was lowest in EHTs cultivated at fixed length (S0, 77.6 ± 2.5% residual force) and correlated with the intensity of stretch conditioning (S3, 64 ± 2.4% residual force, Figure 4). This relationship indicates a parallel enhancement of contraction force and respiratory capacity in progressively stretched tissues.

### Stretch conditioning improves contractility and passive elasticity of EHTs

**Figure 5:**
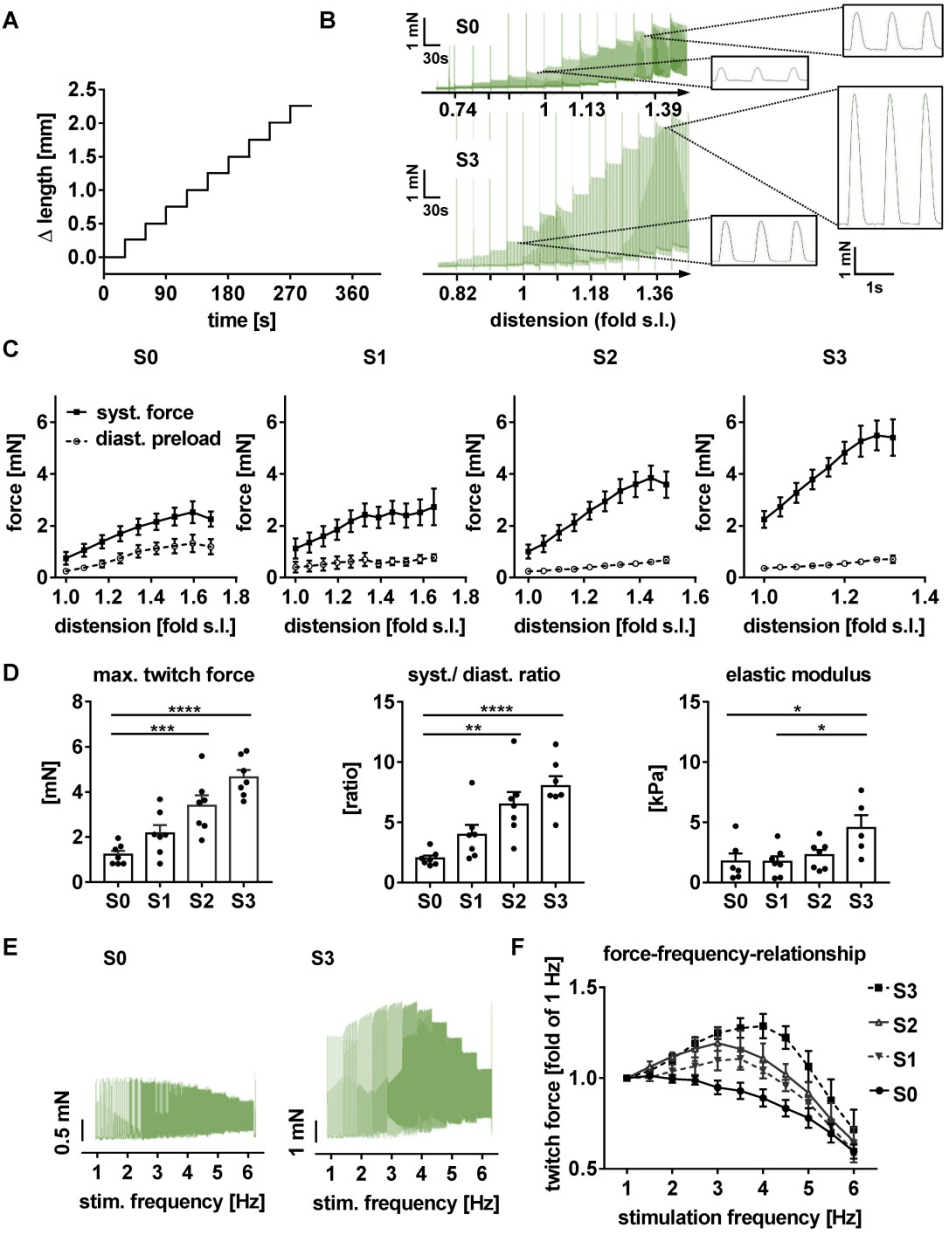
** Stretched EHTs approach myocardial biomechanical properties.** Representative length (A) and force (B) traces demonstrate the response of a contracting EHT to increasing distension under isometric conditions, and represent an intact Frank-Starling relationship. (C) Response of systolic and diastolic forces to distension in stretch-conditioned EHTs (n = 7). (D) Maximum twitch force, ratio of systolic and diastolic force, and elastic modulus after 3 weeks of biomimetic culture with static (S0), low (S1), moderate (S2), or high (S3) stretch (n = 7). (E) Representative force-frequency-relationship of EHTs exposed to static (S0) or high stretch (S3). (F) Force-frequency-relationship in stretch-conditioned EHTs (n = 6-7). (B) and (C): s. l. (slack length). (C, D and F) n = 7 EHTs per group. Two Way ANOVA, Tukey's multiple comparison test vs. static stretch, **p < 0.01, ***p < 0.001, ****p < 0.0001.

After 21 days of biomimetic culture, EHTs were transferred to an organ bath and exposed to acute distension and various pacing rates under isometric conditions. Tissues tolerated elongation up to 1.8-fold slack length and accordingly developed increased diastolic and systolic forces (Figure 5A-D). Both, the contractility response to acute distension and the twitch force at optimum preload increased with the level of stretch conditioning. EHTs stretched at the highest rate not only developed 5.1-fold higher contractility, but also displayed a 2.6-fold increase in elastic modulus (strain/stretch), when compared with tissues exposed to static stretch (S0, Figure 5C). Importantly, conditioning with high stretch enabled EHTs to generate a maximum ratio of systolic by diastolic wall stress of 9.47 ± 0.84 (Figure 5D), thereby approaching the biomechanical characteristics of healthy human myocardium 27.

### Excitation/contraction coupling attains more mature characteristics in stretched EHTs

**Figure 6:**
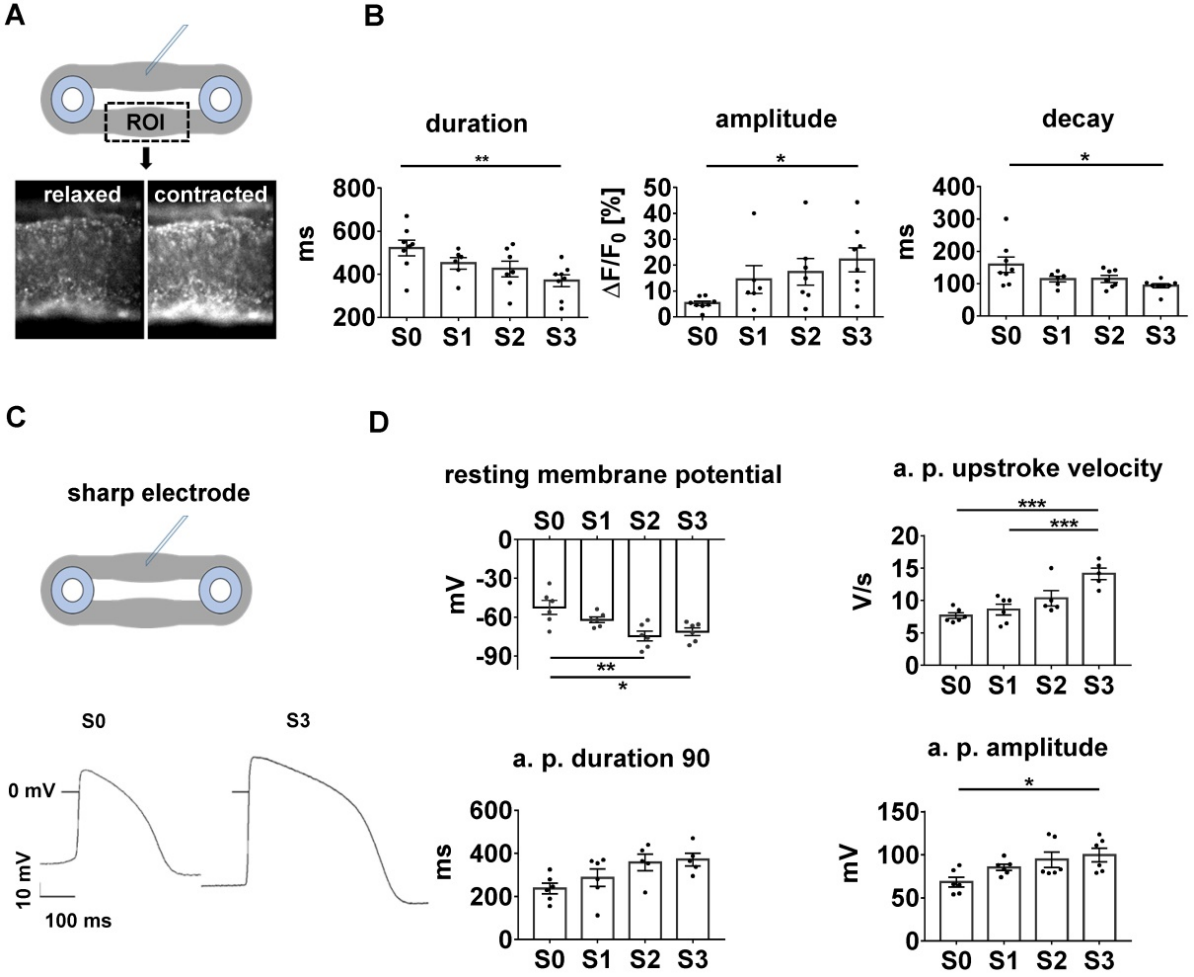
** Parameters of excitation/contraction coupling are improved in stretch-conditioned EHTs.** (A) Snapshots from image analysis of Fluo-4 fluorescence with ROI (region of interest). (B) Quantification of calcium transient duration, amplitude, and exponential decay (τ) in EHTs conditioned with static (S0, n = 8), low (S1, n = 6), intermediate (S2, n = 7), or high (S3, n = 8) stretch. (C) Representative action potentials of EHTs exposed to static or high stretch. (D) Quantification of resting membrane potential, upstroke velocity, action potential (a.p.) duration at 90% repolarization and amplitude (S0-3, n = 6 tissues). (B) One Way ANOVA, Dunnett's multiple comparison test, *p < 0.05, **p < 0.01, (D) One Way ANOVA, Tukey's multiple comparison test, *p < 0.05, **p < 0.01.

With its dependence on membrane potential, Ca^2+^-cycling and a mature ultrastructure, the force-frequency-relationship (FFR) is an integral parameter of electromechanical coupling of cardiomyocytes. EHTs cultured under static stretch exhibited a decreasing contraction force with any increase in pacing rate (negative FFR). In contrast, stretch-conditioned EHTs, particularly S2 and S3, responded with a 1.25-fold force increase to rapid pacing (positive FFR), with optimum performance at 3 and 4 Hz, respectively (Figure 5E-F). To determine the influence of stretch on these functional hallmarks, we measured Ca^2+^-transients in Fluo-4-loaded EHTs at the end of the cultivation period (Figure 6A-B). As expected, higher rates of progressive stretch resulted in increased amplitudes of systolic Ca^2+^ and accelerated Ca^2+^-cycling, as indicated by shorter Ca^2+^-transient durations and time constant (τ) of Ca^2+^-decay (Figure 6B). In separate experiments, we also assessed the electrophysiological responses to stretch application (Figure 6C-D). Intensely stretched EHTs (S3) displayed more negative resting membrane potential (-72.4 ± 3.4 mV vs. -52.5 ± 5.3 mV), higher action potential amplitudes (99.7 ± 7.9 mV vs. 68.3 ± 5.6 mV) and increased upstroke velocities (13.47 ± 3.2 V/s vs. 7.79 ± 0.5 V/s, n = 6) compared to tissues at static stretch (S0). Similar improvements were observed for the contractility response to beta-adrenergic stimulation, which is another hallmark of phenotypic maturation 2,28. Stretch potentiated the positive inotropic and lusitropic response to isoproterenol, indicated by increased contractile force, higher relaxation velocity and shorter contraction duration (Figure 4A).

### Stretch conditioning promotes expression of adult-specific cardiomyocyte genes

The influence of stretch on hiPSC-CM differentiation was furthermore deduced from the mRNA expression of selected marker genes. In human heart development, the expression of the beta-myosin heavy chain (MHC) increases, while that of alpha-MHC decreases during the transition from fetal to adult heart 29, with beta-MHC predominantly being expressed in the ventricle 30. In EHTs, intermediate and high rates of stretch increased the ratio of beta-MHC by alpha-MHC mRNA up to 4.6-fold (p = 0.001, n = 6-13) as compared to EHTs of constant length (Figure S6). Surprisingly, this was paralleled by reduced mRNA expression of brain natriuretic peptide (NPPB, p = 0.008, n = 6-13), which is regarded as a stress-responsive gene 31. Due to the limited impact of stretch on the genes selected for PCR analysis (Figure S6), we obtained a more general picture of gene expression by RNA sequencing. An unbiased selection of genes that are specifically expressed in adult in contrast to embryonic cardiomyocytes 22 was used to evaluate maturation of stretched EHTs. The average transcript abundance of these genes was 2.1-fold higher in stretch conditioned (S3) vs. statically cultured (S0) EHTs (Figure S5A), thus indicating an improvement of cardiomyocyte maturation. Expression of individual genes was analyzed to indicate individual pathways involved in maturation. In this regard, RNA sequencing detected profound induction of genes related to excitation/contraction coupling and oxidative phosphorylation in stretch conditioned tissues (Figure S5B-C). Induction of beta-oxidation (PPARalpha) and suppression of anaerobic glycolysis (LDH) may represent a metabolic switch. Interestingly, specific markers of non-myocyte cell types (e.g. fibroblasts and endothelial cells) were also affected by stretch, thus indicating the importance of cellular interactions that need to be explored in more detail (Figure S5C).

### Stretch induces cellular growth and improves myofibril density and alignment

**Figure 7:**
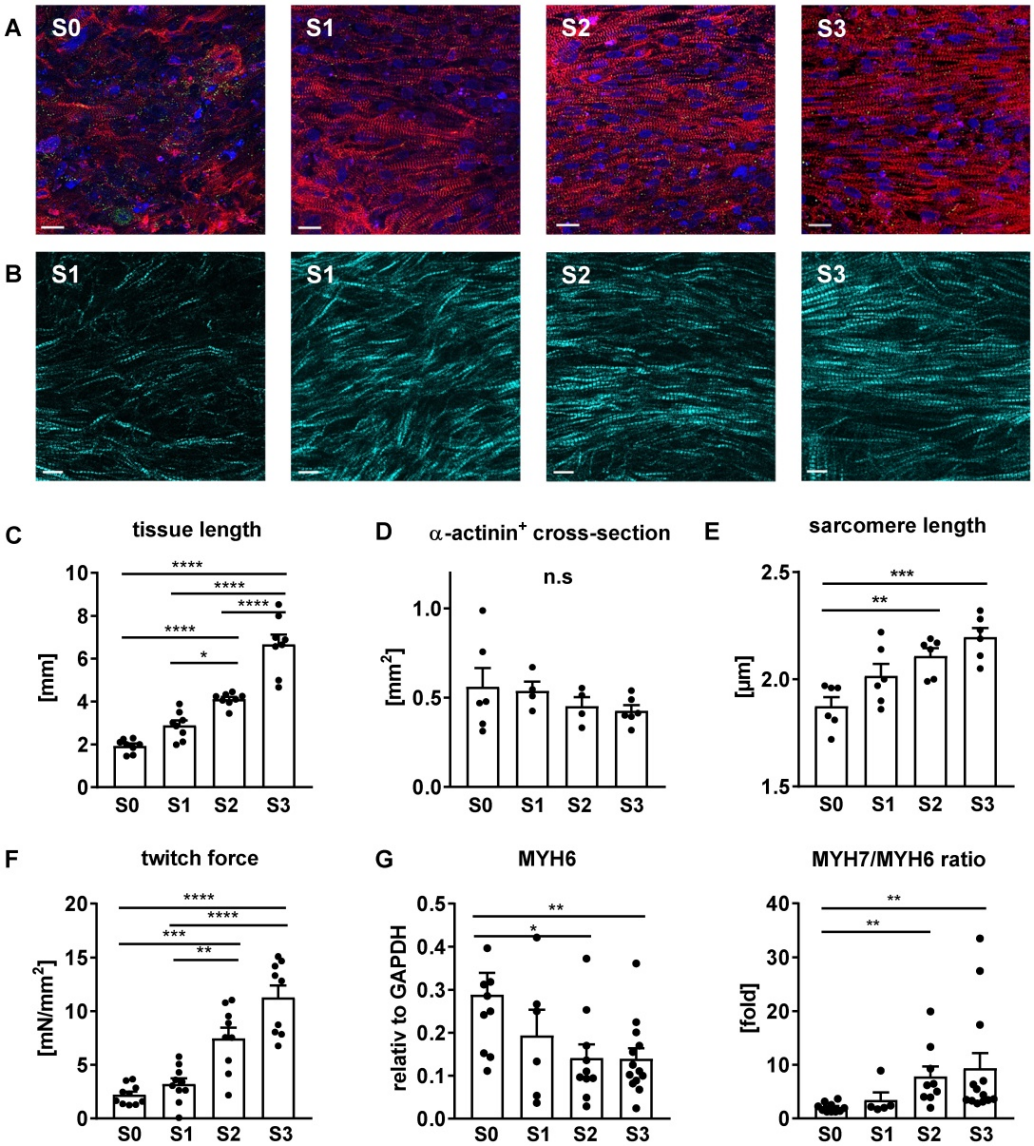
** Myofibril alignment and maturation contribute to stretch-induced gain of contractility.** (A) Immunofluorescent images of EHTs exposed for three weeks to static (S0), low (S1), moderate (S2), or high (S3) stretch. Longitudinal section stained for alpha-actinin (red), connexin43 (green) and DNA (blue). Scale bar: 10 µm (B) Second harmonic generation imaging of stretch-conditioned EHTs detailing alignment and density of myofibrils and sarcomeres. Scale bar: 10 µm (C) EHT slack length after three weeks of culture (n = 8). (D) α-actinin positive cross section after three weeks of culture (n = 6 for S0 and S3, n = 4 for S1 and S2). (E) Sarcomere length after three weeks of culture (mean values of n = 6 tissues). (F) Specific twitch force of differently stretched EHTs after 3 weeks of biomimetic culture (n = 10). (G) Maturation of myosin isoform expression: Quantitative PCR analysis of MYH6 mRNA and MYH7/MYH6 ratio. (C-G): One-way ANOVA, Tukey's multiple comparison test. *p < 0.05, **p < 0.01, ***p < 0.001, ****p < 0.0001.

**Figure 8:**
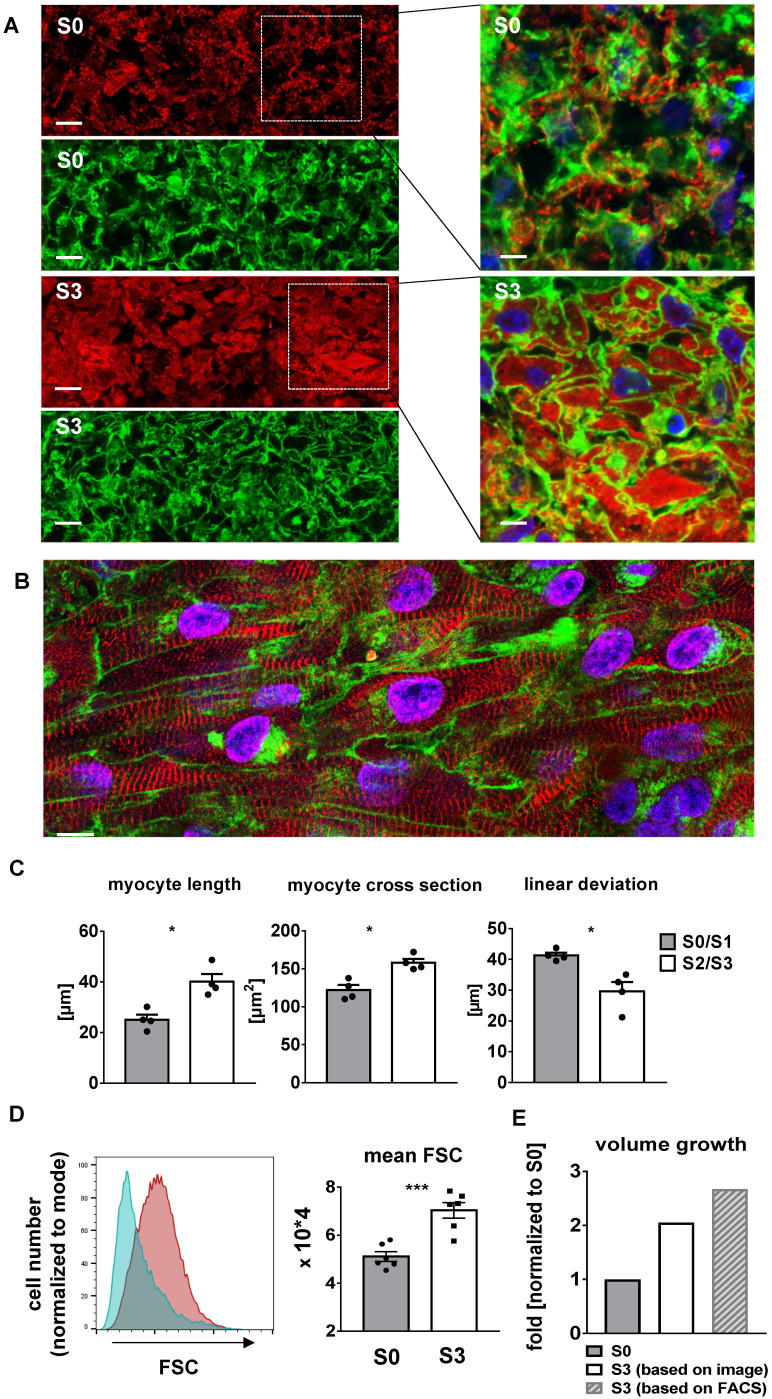
** Progressive stretch promotes growth and linear orientation of hiPSC-CMs.** (A) High magnification cross sections of EHTs conditioned with S0 or S3 stretch, scale bar left: 10 µm, scale bar right: 5 µm. WGA-negative areas correspond to cell-free areas (predominant in S0), or to cytosol cross sections (predominant in S3). (B) High magnification longitudinal section of S3-conditioned EHT, scale bar: 10 µm (C) Morphological parameters of cardiomyocytes and cardiomyocyte alignment in EHTs after low stretch (S0, S1) or high stretch (S2, S3) conditioning (mean values of n = 4 tissues). (D) Representative histogram of forward scatter (FSC) shifting and myocyte volume analysis in terms of mean FSC. Cyan peak indicates FSC of S0 (static stretch, cTnT positive); red peak indicates FSC of S3 (high stretch, cTnT positive). Cell suspensions were stained with cardiac muscle troponin T (cTnT). (E) Schematic diagram of cell volume growth estimation derived from different analyses. (A and B) stained for alpha-actinin (red), WGA (green) and DNA (blue) after three weeks of culture, (C) Mann-Whitney test. (D) n = 6 for S0 and S3. unpaired t-test. *p < 0.05, **p < 0.01, ***p < 0.001, ****p < 0.0001.

Progressive stretch resulted in increased tissue length (Figure 7C), which was closely correlated with the extension of cardiomyocyte length (Figure 8C). An increase in cellular volume of stretched cardiomyocytes was also shown by flow cytometry after tissue dissociation (Figure 8D-E). Since the total actinin ^+^ cross-section area, as well as its contribution to tissue cross-section area, were not altered by stretch conditioning (38-48% among all groups, n = 2-6, n. s.; Figure 7D), the estimated cardiomyocyte volume (mean length multiplied by mean cross-section) was approximately doubled in progressively stretched tissues (S0/S1: 3050 µm^3^; S2/S3: 6270 µm^3^), corresponding to a similar increase in EHT muscular mass (tissue length multiplied by actinin ^+^ cross-section, S0: 1.3 mm^3^, S3: 2.7 mm^3^; Figure 7C-D). Stretch conditioning also improved hiPSC-CM alignment with the direction of force development (Figure S3) and with neighboring cardiomyocytes, as indicated by reduced cell axis dispersion (Figure 8C). Stretch also extended the sarcomere length from 1.87 ± 0.1 µm (S0) close to physiological levels (S3: 2.19 ± 0.1 µm; Figure 6D). These features may contribute to the physiological force/length dependency of stretch-conditioned EHTs, which closely resembled the Frank-Starling mechanism (Figure 5A-D). Ultrastructural investigation of alpha-actinin ^+^ regions by confocal and second harmonic generation microscopy revealed large numbers of cardiomyocytes with densely packed and linearly arranged cross striations in stretched tissues (S1-S3) (Figure 7A-B; Figure 8A-B), whereas cardiomyocytes demonstrated scattered distribution and sparse cross striations in non-stretched tissues (S0). High magnification imaging revealed an increased fractional area of alpha-actinin ^+^ myofibrils in myocyte cross-sections, and tighter assembly of cardiomyocytes in progressively stretched vs. static stretched EHTs (Figure 8A). The maturation of myofibril density and structure, along with tight mechanical cellular coupling may be regarded as the structural basis of the contractility increase brought about by progressive stretch conditioning.

## Discussion

Cardiac organogenesis is governed by numerous factors whose close *in vitro* simulation is considered to be a keystone to artificial tissue generation. Biomechanical conditions have been demonstrated to be of eminent importance for the maturation of postnatal and of stem cell-derived cardiomyocytes 32, but their deployment is technically demanding. In particular, hypertrophy of cardiomyocytes, a hallmark of postnatal cardiac growth, requires size adjustment and guidance by appropriate biomechanical forces. In order to generate such conditions, we utilized a recently developed bioreactor that had been shown to support the survival and continuous contractile performance of human adult myocardium *in vitro* for up to 4 months 18. This bioreactor not only provided biomimetic conditions well established in cardiac tissue maturation, such as defined compliance and electrical stimulation, but also was able to expose the artificial tissues to defined passive forces. With this approach, we could show that EHTs generated from hiPSC-CMs respond to progressive stretch with elongation and linear alignment of cardiomyocytes, enhanced density and structure of myofibrils, and with excitation-contraction characteristics that approached those of adult human myocardium, including positive force-frequency and force-stretch relationships.

After birth, cardiomyocytes quickly lose the capability of cell division 33,34, rendering hypertrophy the predominant cellular process for the postnatal 30- to 40-fold increase in volume of individual myocardial cells 34. In this phase, myocardial growth reflects an adaptive response that enhances size and performance of the heart, and thereby contrasts with the pathological hypertrophic remodeling induced by excessive load 35. Therefore, appropriate mechanical loading can be expected to be a requirement for terminal differentiation and maturation of stem cell-derived cardiomyocytes. This notion has been confirmed in several studies that investigated various implementations of mechanical loading on 2D or 3D artificial heart tissues 36-38, such as static strain and cyclic stretch 39-45. The biological actions of cyclic stretch, however, differ fundamentally from those of progressive distension, as applied in the present study. Due to the fixed size of the final constructs, passive cyclic stretching will induce corresponding strain in the tissues without enlarging its dimensions, whereas progressive distension provoked growth of the EHTs with increase in cardiomyocyte size (Figure 8D) and no consistent increase in strain (S1 - 2 groups, Figure 3D). These biomechanical conditions provoke a 5.1-fold increase of contractility (S3, Figure 7F), doubling of tissue length (Figure 7C), and normalization of sarcomere distances (Figure 7D). In addition, we identified a limit of daily extension that did not enhance the diastolic preload to levels exceeding unstretched tissues, i.e. that was fully compensated by longitudinal growth (S2 = 0.16 mm/day). As such, the presented technology paves the way to the generation of larger myocardial tissue, since the concept may easily be up-scaled, and it may be particularly useful when applied to tissues with regenerative capacity, e.g., cardiomyocyte progenitors.

As a baseline reference of stretch conditioning, we chose conditions of EHT generation and maintenance that combined several recently reported maturation promoters, such as electrical stimulation 2,9,46 and optimized medium 22. We applied continuous pacing at normal heart rate (1 Hz), and observed deceleration of spontaneous excitation, regular 1:1 pacing at 1 Hz, and progressive decline of stimulation threshold (Figure S4) as indicators of electrical maturation. Likewise, we utilized a serum-free EHT maintenance medium 22 and hormonal supplements with clear evidence for further support of maturation 47,48. Importantly, our standard culture conditions included continuous mechanical agitation of the culture medium in order to enhance O_2_ supply by convective transport. Respective measures of medium agitation, perfusion, or oxygenation are required for the maintenance of adult multicellular preparations 18 and have been shown to dramatically improve the structure and performance of iPSC-derived cardiac bundles 8. The real-time contractility measurements of our study provide direct evidence that the O_2_ demands of highly contractile EHTs are not met by stationary incubation, and are even more pronounced in stretch-conditioned muscle (Figure 4). The higher demand of oxygen in stretch-conditioned EHTs is also reflected by an increased density and more linear network of mitochondria (Figure 4C). The induction of cellular growth in combination with highly optimized baseline conditions may explain why functional features of stretch-conditioned EHTs, such as specific contractility and FFR, approach physiological characteristics, whereas some structural features, such as the formation of t-tubuli or the confinement of connexins to intercalated discs, fall short of these improvements (Figure 7A). The artificial process of differentiation, imperfect interactions with extracellular matrix and non-myocyte tissues as well as the absence of various hormones may account for this.

Stretch conditioning remarkably improved the biomechanical properties of EHTs. With the help of continuous monitoring, a steady increase of contractility was observed in static, but even more in stretched preparations. The contractility of stretch-conditioned EHTs (11.3 mN/mm²) exceeded the systolic performance even of the most recently reported preparations (< 5 mN/mm²) 2,9,19,22, with one particular exception (23 mN/mm²) 11,12. As such, stretch-conditioned EHTs almost reached the active force development of adult human heart (15.1 mN/mm²) 49, a performance that certainly encompasses the demonstrated structural and functional improvements, e.g. of myofibril density, cell size, linear alignment, electrical excitation, response to adrenergic stimulation, sensitivity to hypoxia, and Ca^2+^-cycling.

Improvements in cell size (an indicator of maturation 50), and in sarcomere length (2.2 μm, identical to adult human ventricular myocytes 50) paralleled the stretch induced increase in contractility. However, the cell size of adult human cardiomyocytes was not reached within three weeks of culture. Morphometry and flow cytometry of the cardiomyocytes confirmed that tissue elongation was not brought about by reversible elastic strain but rather by longitudinal cellular growth. Despite increments in tissue length and volume, no significant increase in wall stress was detectable at low and intermediate rates of distension (S1, S2). Under these circumstances, the applied progressive stretch directly corresponded to cellular growth. Only the high rate of stretching increased diastolic tension to a measurable extent, thereby generating an additional growth stimulus. This, however, was not sufficient to stabilize diastolic forces, i.e. diastolic force rose with each increment in distension, which sometimes resulted in EHT tearing. Thus, the highest rate of distension probably exceeded the maximum rate of cardiomyocyte growth.

Similar to contractility, progressive distension also promoted the generation of passive stiffness of EHTs, at the highest rate resulting in an elastic modulus (4.5 kPa) approaching that of healthy human or porcine myocardium (5-7 kPa 51, 5.8 kPa 52, 5-7 kPa 53). While only a small fraction of this elasticity (1.5 kPa) is generated physiologically by the extracellular matrix 52, engineered tissues frequently exceed this mechanical contribution of the matrix, since cultured cardiomyocytes have been reported to profit from attachment to scaffolds that simulate the stiffness of total tissue 54. The high stiffness of the matrix, however, fosters focal adhesion of cardiomyocytes to the matrix and may impede the development of intercellular force transmission 55. Since our model relies on the generation of effective mechanical load by external forces (diastolic distension and elastic contraction), we sought to avoid the possible pathophysiological influence of excessive matrix by using low amounts of matrix constituents of EHT assembly. This strategy enabled the development of active and passive forces of EHTs at an adequate ratio of 9 under elastic conditions in BMCCs, and of 9.5 at optimum preload under isometric conditions in the organ bath. These properties correspond well with the ratio of peak systolic to end-diastolic wall stress of adult human myocardium (10.2 ± 1.7) 27, thus indicating that EHTs share similar abilities to translate external distension into changes of sarcomere length.

As an indicator for progression along the lines of cardiac development, a shift in the expression of alpha-MHC to beta-MHC was observed in stretched EHTs. While other genes selected for rt-PCR analysis provided little further evidence of maturation, a global assessment of gene expression by RNA sequencing revealed a prominent up-regulation of a preselected group of adult-specific cardiomyocyte genes in stretched EHTs. Since the latter approach refers to a typical cardiomyocyte fingerprint of gene expression, and is well accepted for the assessment of organ development, we may state that advancements to a more mature transcriptional program are evident in stretch-conditioned hiPSC-CMs. Stretch-dependent components of gene expression could be identified in pathways of excitation/contraction coupling, metabolism, and in non-myocyte cell types.

Surprisingly, we observed downregulation of brain natriuretic peptide (BNP) expression at all levels of external distension which seems to contrast with the mechanosensitive induction of BNP expression in heart failure, as well as in EHTs contracting under isometric or low compliance conditions 7,41,56. Such biomechanical conditions differ from the exclusively elastic contractions that iPSC-CMs perform in BMCC culture, which obviously avoid mechanical overload even under repeated distensions. Our measurements of diastolic preload agree with this notion since they demonstrate no (S1, S2) and only minor (S3) increase of diastolic forces at different rates of progressive stretch. Overall, these EHT data suggest that systolic rather than diastolic stress might be the dominant stimulus for BNP release, thereby confirming respective observations in human subjects 57,58. EHTs under defined biomechanical load may therefore be a suitable model to study the yet unknown mechanotransduction of natriuretic peptides.

With the aim to develop a versatile protocol for EHT formation, and to focus on the investigation of biomechanical stimuli, we omitted some more complex techniques of EHT engineering. In particular, a standard small-molecule protocol was employed for inducing differentiation of a generic iPSC line, yielding 69% cardiomyocyte purity (alpha-actinin and cTnT double-positive cells). With no further purification or modification (e.g., fibroblast co-cultivation), the full cell population was suspended in collagen hydrogel, and was assembled on a filter surface. This modification of primary construct formation was introduced in order to accelerate tissue solidification and to minimize construct shrinkage. The approach also avoided the use of a pre-structured matrix scaffold, and thus relied on the primary collagen/laminin hydrogel and on endogenous synthesis or remodeling of more physiological matrix components, by mesenchymal cells that potentially existed in the non-myocyte fraction. In this way, the formation of a primary tissue was possible at high cell density, which promoted close cellular contacts, rapid resumption of rhythmic activity, and enabled an early commencement of the stretching protocol.

A limitation of the study is the fact that only one hiPSC line was tested at a fixed time point of differentiation to evaluate the effects of progressive stretch on maturation. However, different maturation approaches with several other hiPSC lines led to similar results 37 and therefore the results of our approach seem to represent general responses. A second limitation may be seen in the fact that the highest stretch rate (S3), although inducing the greatest increase in contraction force, eventually caused EHT tearing, and failed to extend the cardiomyocyte length to normal dimensions. As such, even higher stretch rates are likely to be not feasible with this culture assembly conditions. This may be an inevitable limitation imposed by the short duration of the maturation period. Most likely, however, limitations in growth may also reflect shortcomings in hormonal growth stimuli or nutritional deficiencies. We suggest that a combination of progressive stretch with the most advanced heart tissue engineering protocols, including fibroblast supplementation, hormonal stimulation, and extended culture time, may be required to develop the full maturation potential of stem cell-derived cardiomyocytes.

## Conclusion

This study presents a novel approach to induce maturation and growth of engineered myocardium by progressive stretch simulating the increase in mechanical load during postnatal heart development. Within only three weeks of culture, EHTs improved in size, structure, and contractility, and displayed features of mature excitation-contraction coupling. As such, the integrated biomimetic approach not only facilitates the generation of highly mature human EHTs, but may also shed light on the biomechanics of heart development and may raise the bar for the generation of therapeutically relevant tissue constructs.

## Supplementary Material

Supplementary figures and tables.

## Abbreviations

AP: action potential
BNP: brain natriuretic peptide
BMCCs: biomimetic cultivation chambers
Ca^2+^: calcium
cTNT: cardiac troponin
EHT: engineered heart tissue
FFR: force-frequency-relationship
GAPDH: glyceraldehyde-3-phosphate dehydrogenase
hiPSC-CMs: human induced pluripotent stem cell-derived cardiomyocytes
iPSC: induced pluripotent stem cell
LDH: lactate dehydrogenase
MHC: myosin heavy chain
PPARalpha: peroxisome proliferator activated receptor alpha
RMP: resting membrane potential
SD: standard deviation
SEM: standard error of the mean
