# Supplementary material for: Progressive stretch enhances growth and maturation of 3D stem-cell-derived myocardium
Source: Theranostics. 2021 Apr 15;11(13):6138–53. doi: 10.7150/thno.54999 (PMC8120210; doi:10.7150/thno.54999)
Supplement: Supplementary file 1 — Supplementary figures and tables. [file thnov11p6138s1.pdf]

SUPPLEMENTARY MATERIALS

Progressive stretch enhances growth and maturation of 3D stem-cell-derived myocardium

Kun Lu[1], Thomas Seidel[2], Xiaochun Cao-Ehlker[1], Tatjana Dorn[3], Aarif Mohamed Nazeer Batcha[4,5], Christine Maria Schneider[3], Marie Semmler[1], Tilmann Volk[2], Alessandra Moretti[3,6], Andreas Dendorfer[1,6], Roland Tomasi[1,7]

[1] Walter-Brendel-Centre of Experimental Medicine, University Hospital, Ludwig-Maximilians-University München, Munich, Germany

[2] Institute of Cellular and Molecular Physiology, Friedrich-Alexander University Erlangen-Nürnberg, Erlangen, Germany

[3] First Department of Medicine, Cardiology, Klinikum rechts der Isar, Technical University München, Munich, Germany

[4] Institute for Medical Information Processing Biometry and Epidemiology, Ludwig-Maximilians-University München, Munich, Germany

[5] DIFUTURE (Data Integration for Future Medicine), Ludwig-Maximilians-University München, Munich, Germany

[6] DZHK (German Center for Cardiovascular Research), Partner site Munich Heart Alliance, Munich, Germany

[7] Clinic of Anaesthesiology, University Hospital, Ludwig-Maximilians-University München, Munich, Germany

Corresponding authors:

Andreas Dendorfer & Roland Tomasi shared joint senior authorship. Walter-Brendel-Centre of Experimental Medicine, University Hospital, Ludwig-Maximilians-University München, Marchioninistr. 68, 81377 Munich, Germany, E-mail: andreas.dendorfer@med.uni-muenchen.de, roland.tomasi@med.uni-muenchen.de

Supplementary methods

Flow cytometry

The cell suspension used for EHT generation was tested for purity of hiPSC-derived cardiomyocytes by FACS analysis. For intracellular staining of cardiac troponin T (cTnT) and alpha-actinin, 0.5×10^6 dissociated cells were suspended in 15 mL 4% paraformaldehyde and fixed for 15 min. After centrifugation (200 xg, 5 min, 4 °C) cells were permeabilized in PBS with 1% Triton X-100 and washed twice again with PBS containing 1% BSA (Sigma). Cells were then stained 45 min (4 °C) with the unconjugated primary antibodies targeting cTnT (ab45932, Abcam) and alpha-actinin (A7811, Sigma Scientific) in FACS buffer consisting of PBS with 1% fetal bovine serum (FBS, Gibco). After primary antibody removal with PBS containing 1% BSA, cells were incubated with Alexa Fluor 488 anti-Rabbit IgG (1:500; Invitrogen A-21441), and Alexa Fluor 546 anti-Mouse IgG (1:500; Invitrogen A-11030) diluted in PBS with 1% BSA for 30 min at 4 °C. Cells were then washed twice with PBS and analyzed using a FACS Canto II flow cytometer (BD Biosciences, USA) and the FlowJo software (Treestar Inc., USA).

For determination of cardiomyocyte volumes, the cell suspension of dissociated EHTs was subjected to FACS analysis. EHTs were mechanically reduced to small pieces which were incubated on a thermostated shaker (20 min, 37 °C) with dissociation solution (collagenase II 2,2 mg/mL, Sigma Aldrich and TrypLE Select Enzyme, Gibco). After further mechanical disruption by pipetting, undissolved tissue strips were removed, and the cell suspension was stained for cardiac troponin T (cTNT) as described. Data shown are representative of five independent experiments.

Calcium handling

To assess calcium handling, EHTs within BMCCs were loaded with 5 µmol/L Fluo-4, AM (Invitrogen) under electromechanical stimulation at 1 Hz for 30 minutes at 37 °C, followed by an additional 15 min washout period after medium exchange. Videos were acquired on an inverted microscope during continuous stimulation at a rate of 50 frames/s, using a Rolera EM-C2 camera (Teledyne QImaging, Canada), and data were acquired with Micro-Manager Studio Version 1.4 (University of California San Francisco, USA). Quantitative data were extracted with ImageJ (National Institute of Health, USA), and calcium dynamics were analyzed with WinEDR v3.8.6 (University of Strathclyde, UK). For each experiment, several regions of interest were selected for calculation of the mean fluorescence intensity. The signal was normalized to the diastolic background fluorescence. Calcium transient duration, amplitude, and calcium decay rate were measured.

Simulated hypoxia

For induction of tissue hypoxia, the standard condition of medium agitation (tilting of BMCCs by a 12° angle at 1 Hz) was stopped for a 2 min interval. Electrical stimulation at 1 Hz and contraction force recording was continued throughout.

Isometric contraction performance

Contractility of the EHTs were determined under isometric conditions in horizontal organ baths (Mayflower, Hugo Sachs Elektronik, Germany) as previously described [1]. In brief, EHTs were attached to isometric force transducers (F30, HSE) with preload adjusted to 1 mN, and continuously perfused with oxygenated Tyrode solution (1.8 mmol/L $CaCl_2$ and 23 mmol/L bicarbonate, pH 7.4) for at least 10 min prior to any measurement. Electrical stimulation (1 Hz, 3 ms pulse width, 2-fold stimulation threshold) was initiated immediately.

The maximum twitch force was determined under optimum preload. For determination of length-tension relationship, tissues were distended in steps of 0.25 mm. Baseline (slack length) was defined as the longest extension that produced no diastolic force. Passive and active forces were recorded continuously and analyzed 15 seconds after each distension step. The force-frequency relationship was determined by the stepwise increase of stimulation frequency from 1 Hz to 6 Hz. The elastic modulus was calculated from the slope of diastolic force at the distension preceding the condition of the maximum contractility. The slope of diastolic force was related to the length and actinin$^+$ cross-section area of the tissue.

Isoproterenol response

Isoproterenol was dissolved in Tyrode solution and added to the organ bath at a 1:1000 v/v dilution. The contraction force was continuously recorded. Drug-induced changes in the generated force, relaxation rate, and contraction duration were determined.

Action potential recording

Action potentials (APs) were recorded with standard sharp microelectrodes in intact EHTs after 21 days of culture, as previously described [2]. Tissues were mounted at relaxed length in a thermostated chamber (37 °C), and superfused with HEPES-buffered Earle' s-salt solution. Field stimulation was performed at 1 Hz with monopolar impulses of 3 ms duration at excitation threshold intensity. Membrane potential was amplified with a BA-01X amplifier (NPI electronic, Germany), and recorded with PowerLab 4/20 (AD-Instruments, Dunedin, New Zealand). Resting membrane potential (RMP), amplitude, upstroke velocity, and AP duration were analyzed with standard functions of LabChart software (ADInstruments).

Quantitative PCR analysis

RNA extraction was performed using the RNeasy Mini Kit (Qiagen). RNA integrity number (RIN) was determined with the Simplinano Spectrophotometer (Biochrom). Subsequently, equal amounts of RNA were reversely transcribed using the QuantiTect Reverse Transcription Kit (Qiagen) following the manufacturer's instructions. All qRT-PCR analyses were performed in duplicate on a StepOnePlus Real-Time PCR System (Applied Biosystems). Cycling conditions were as follows: holding stage 95 °C for 10 min, cycling stage 40 cycles at 95 °C for 15 s, 60 °C for 1 min, and 72 °C for 15 s. Data analysis was carried out using the fold change normalized to glyceraldehyde-3-phosphate dehydrogenase (GAPDH) gene expression. A primer list is provided in Table S2.

Immunostaining

After washing three times with PBS, whole tissues were prepared for cryosectioning. First, EHTs were placed at 4 °C overnight in 1 mL of 30% sucrose solution in PBS. The next day, we embedded the EHTs in a 7.5% gelatin, 30% sucrose solution. A freezing bath was prepared by dropping several small pieces of dry ice into a bath of isopentane to reach a temperature between -50 ° and -30 °C. The block of gelatin was immersed into the cold bath and allowed to freeze for 2 min. Sections were cut for immunostaining in a standard cryostat. The sectioned tissues were washed with PBS for 30 minutes, permeabilized with 1% Triton X-100 in PBS for 60 min, and then incubated in blocking solution (3% bovine serum albumin and 1% fetal calf serum in PBS) for 1 hour. The primary monoclonal anti-α-actinin antibody (sarcomeric, 1:100, Sigma-Aldrich) in antibody dilution buffer (Supplement Table S1) was incubated overnight 4 °C. The next day, samples were incubated with goat anti-mouse IgG (H+L) highly cross-adsorbed secondary antibody, Alexa Fluor 546 (Invitrogen), at 1:100 in antibody dilution buffer for 2 hours. Tissues were washed three times for 10 min in PBS and

subsequently incubated with DAPI (2 µmol/L, Invitrogen), and wheat germ agglutinin (WGA) conjugated to CF633 (BioTium CF) at 40 µg/mL overnight at 4 °C. Sections were mounted with Fluoromount-G (Invitrogen).

References

1. Brandenburger M, Wenzel J, Bogdan R, Richardt D, Nguemo F, Reppel M, et al. Organotypic slice culture from human adult ventricular myocardium. Cardiovasc Res. 2012; 93: 50–9.

2. Fischer C, Milting H, Fein E, Reiser E, Lu K, Seidel T, et al. Long-term functional and structural preservation of precision-cut human myocardium under continuous electromechanical stimulation in vitro. Nat Commun. 2019; 10: 117.

3. Tiburcy M, Hudson JE, Balfanz P, Schlick S, Meyer T, Chang Liao ML, et al. Defined engineered human myocardium with advanced maturation for applications in heart failure modeling and repair. Circulation. 2017; 135: 1832–47.

Table S1. Media formulations

Medium		
**Medium name**	**Supplement**	
EHT culture medium (base medium IMDM, Gibco, No. 12440061)	B27 minus Insulin (Gibco, A1895601), %	2
	MEM non-essential amino acids solution (Gibco, 11140050) , %	1
	penicillin, U/mL	50
	streptomycin, µg/mL	50
	dexamethasone(Sigma Aldrich, D4902), µmol/L	1
	IGF-1 (PEPROTECH ,100-11), ng/mL	100
	FGF-2 (PEPROTECH, 100-18B), ng/mL	10
	VEGF165 (PEPROTECH, 100-20), ng/mL	5
	TGF-ß1 (PEPROTECH,100-21), ng/mL	5
EB6 medium (base medium DMEM/F-12, Gibco, No. 11320033)	MEM non-essential amino acids (Gibco, 11140050), %	1
	penicillin, U/mL	50
	streptomycin, µg/mL	50
	fetal bovine serum,% FBS Premium South America; PAN Biotech	6 (v/w)
	beta-mercaptoethanol, mmol/L	0.1

IMDM indicates Iscove's Modified Dulbecco's Medium; DMEM/F-12 indicates Dulbecco's Modified Eagle Medium/Nutrient Mixture F-12; EHT, engineered heart tissue; FGF-2, fibroblast growth factor-2; IGF-1, insulin-like growth factor 1; TGF-β1, transforming growth factor-β1; VEGF165, vascular endothelial growth factor 165.

Table S2. Primers for qPCR

Name	5'Sequence	3'Sequence
MYH6	GCCCTTTGACATTCGCACTG	GGTTTCAGCAATGACCTTGCC
MYH7	GTATGGCAAGACAGTGACCG	CGATCCTTGAGGTTGTAGAGC
SCN5A	CTCTATGGCAATCCACCCCAAG	CTGAGGACATACAAGGCGTTGG
KCNJ2	GTAAAGTCCACACCCGACAAC	CCAGCGAATGTCCACACAC
ACTN2	ATGGCCTTGGACTCTGTGC	GGTGTTCACGATGTCTTCAGC
NRAP	GGCAAATGAGAGAGCCTATTGG	CCTGGGTTGCTCATAGTCTTC
MYOM2	CCTGGGAGAGACACACATTTG	CTTTGTACCACTGCACCACG
COL4A5	CCAGGAATACCAGGTCCTAAAGG	GGTCACCTGGAAGACCTACATC
MYL4	CACTGCCGACCAGATTGAAGAG	CTCGGCATTGGTAGGGTTCTG
VCAM1	GAGGAGTGAGGGGACCAATTC	CTAGGGAATGAGTAGAGCTCCAC
SMPX	CCAATGTTAGAGCCATCCAGGC	GAACACCCTCCTCCACTTCAG
TTN	GGATATGAGGCATCAATAGCCGG	CTCACTGGGCTTCACAGTAGG
NPPB	TGGAAACGTCCGGGTTACAG	CTGATCCGGTCCATCTTCCT
CACNA1C	GCCGCTGCAGGAGAGTTTTA	CCCACATGTGCAAGACCACA
SERCA	CATCAAGCACACTGATCCCGT	CCACTCCCATAGCTTTCCCAG

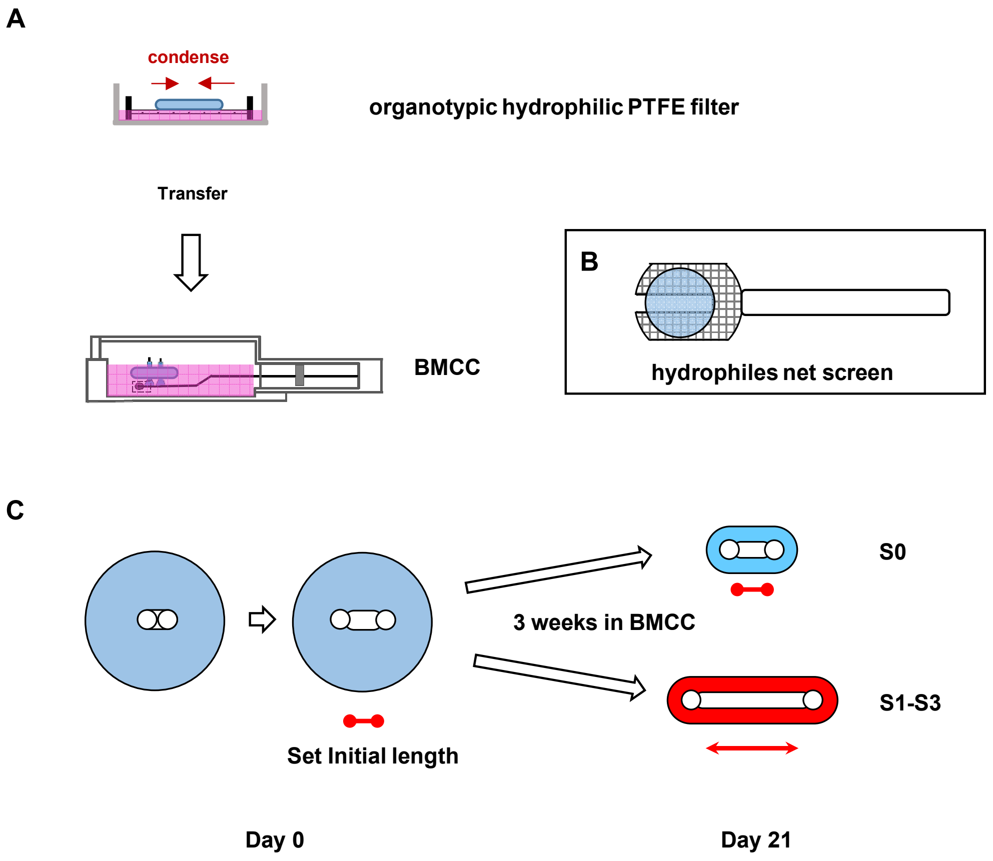

Figure S1. Transfer of the primary EHT and implementation of stretch.
(A) Transfer of the disc-shaped primary assembly of hiPSC-CMs to a biomimetic culture chamber (BMCC) using a custom-made nylon net (B) for support of the fragile tissue. (C) Illustration of the stretching process, implying a daily extension of EHTs by adjustment of the EHT holding posts to a successively increasing distance. Red arrows indicate the direction of preload forces.

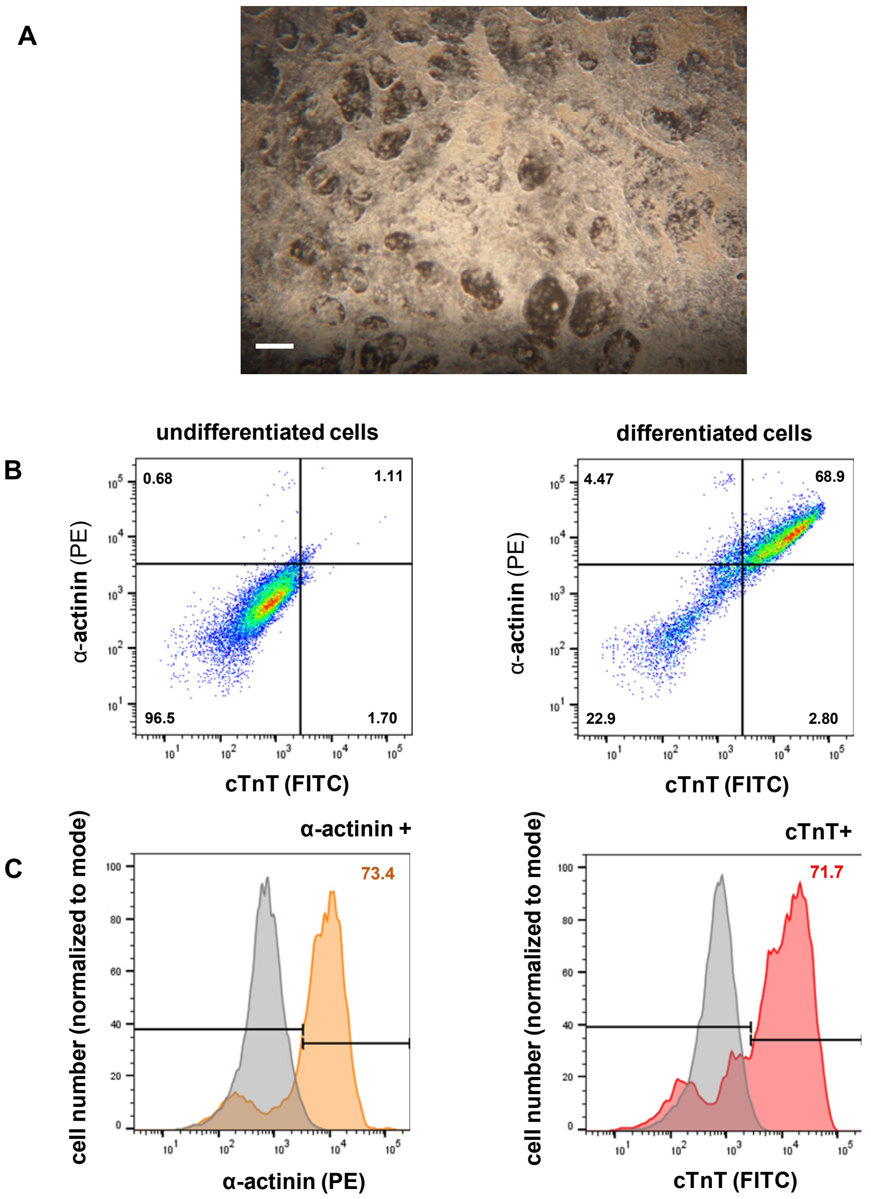

Figure S2. Characterization of hiPSC-CMs used for EHT generation. (A) Confluent cultures of beating hiPSC-cardiomyocytes, scale bar: 300 µm (B) Flow cytometry of undifferentiated hiPSC (MRIi004-A) and differentiated hiPSC-CMs as used for EHT fabrication. Cell suspensions were stained with cardiac muscle troponin T (cTnT) and alpha-actinin. cTnT and alpha-actinin double positive cells were considered to be cardiomyocytes. (C) Representative distribution of alpha-actinin and cTnT in hiPSC-CM (orange and red peak, respectively) and in undifferentiated MRIi004-A stem cells (grey peaks).

A

B

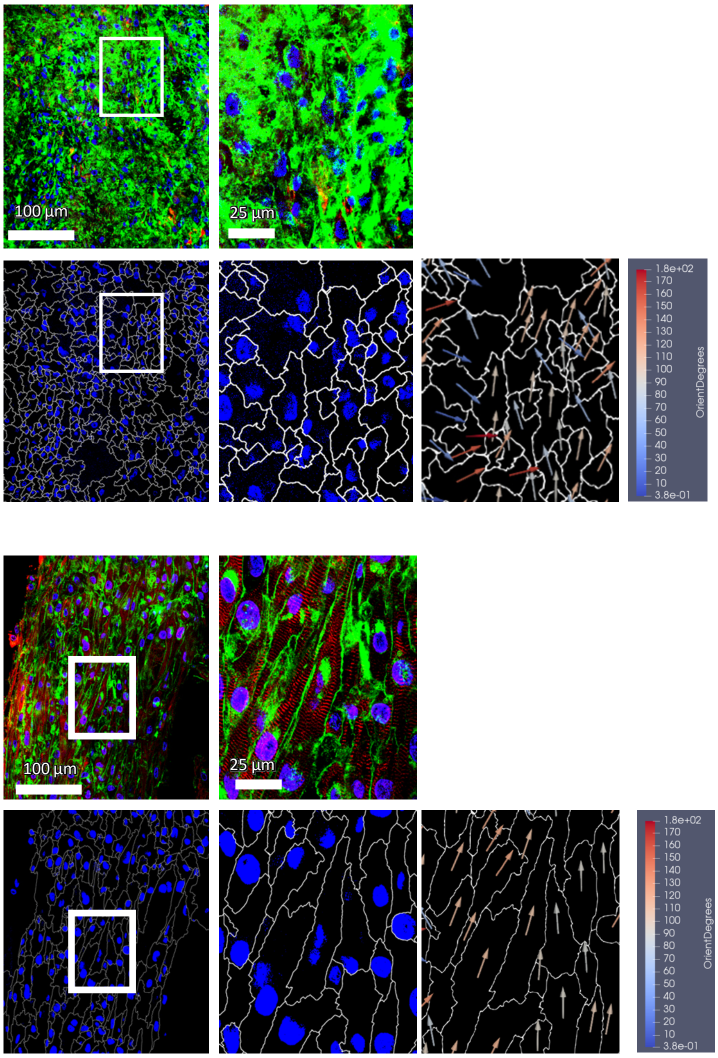

Figure S3. Computerized analysis of structure and alignment of hiPSC-CM. Representative immunofluorescent images obtained with confocal microscopy after three weeks of culture under conditions of (A) static stress (S0) and (B) high stretch (S3). Top panels show longitudinal sections with magnified views of the boxed regions, stained with WGA for extracellular matrix and cell membranes (green), for alpha-actinin (red), and with DAPI for nuclei (blue). Bottom panels show the corresponding cell borders (white) and nuclei (blue) detected by automated image processing. Arrows indicate the orientation of cardiomyocyte main axes.

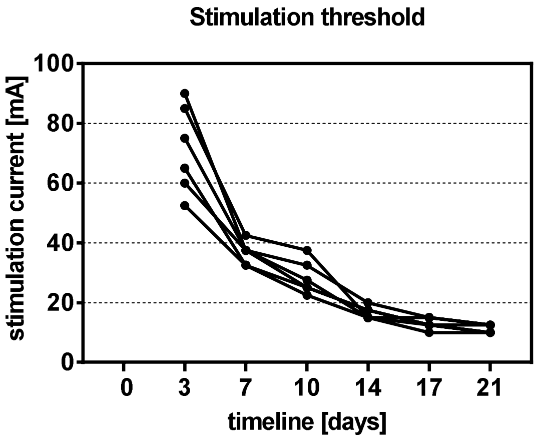

Figure S4. The threshold for EHT pacing decreases during culture. The stimulation current required for electrical excitation of EHTs progressively declined during the first 3 weeks of culture. No difference was detected between the various stretch conditions. The graph displays exemplary data from EHTs exposed to different stretch conditions (S0 - S3, n = 6).

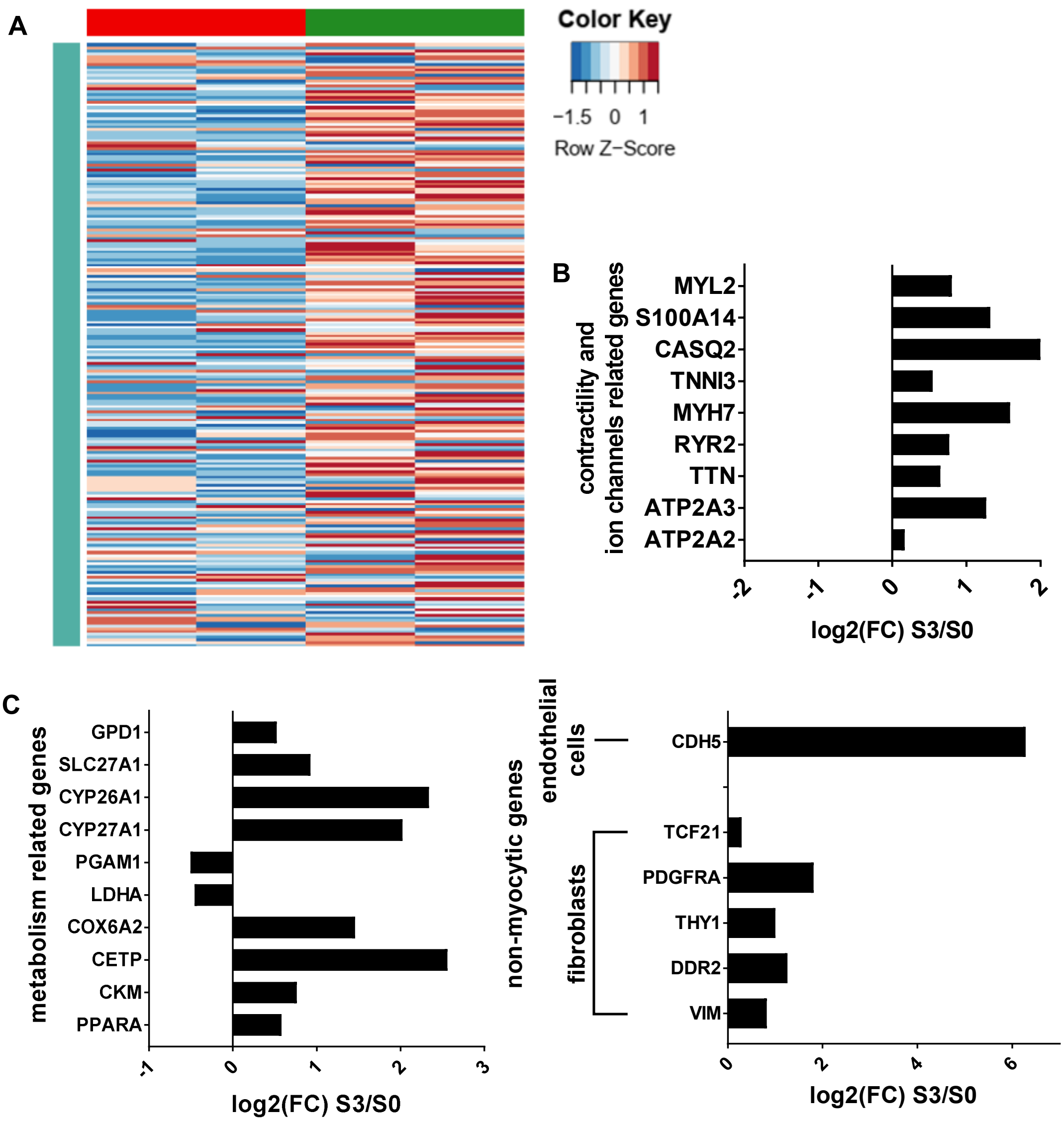

Figure S5. RNA sequencing reveals cellular maturation of hiPSC-CM, and alterations of non-myoyte gene expression in stretch-conditioned EHTs.
(A) Relative RNA expression in EHTs of 218 genes identified as specifically expressed in adult vs. embryonic cardiomyocytes [3]. The heatmap displays the relative alteration of mRNA abundance in statically stretched EHTs (upper red bar) and in intensely (S3) stretched EHTs (upper green bar). (B) Ratio of mRNA expression in stretched (S3) vs. statically cultured EHT of genes related to cardiac contractility and ion channels. (C) Relative mRNA expression in stretched (S3) vs. statically cultured EHTs of genes relevant for energy metabolism (left panel), or indicative of stretch responses of non-myocytic cell types. Data are presented as means of log2 values of relative mRNA abundance. n = 2 experiments.

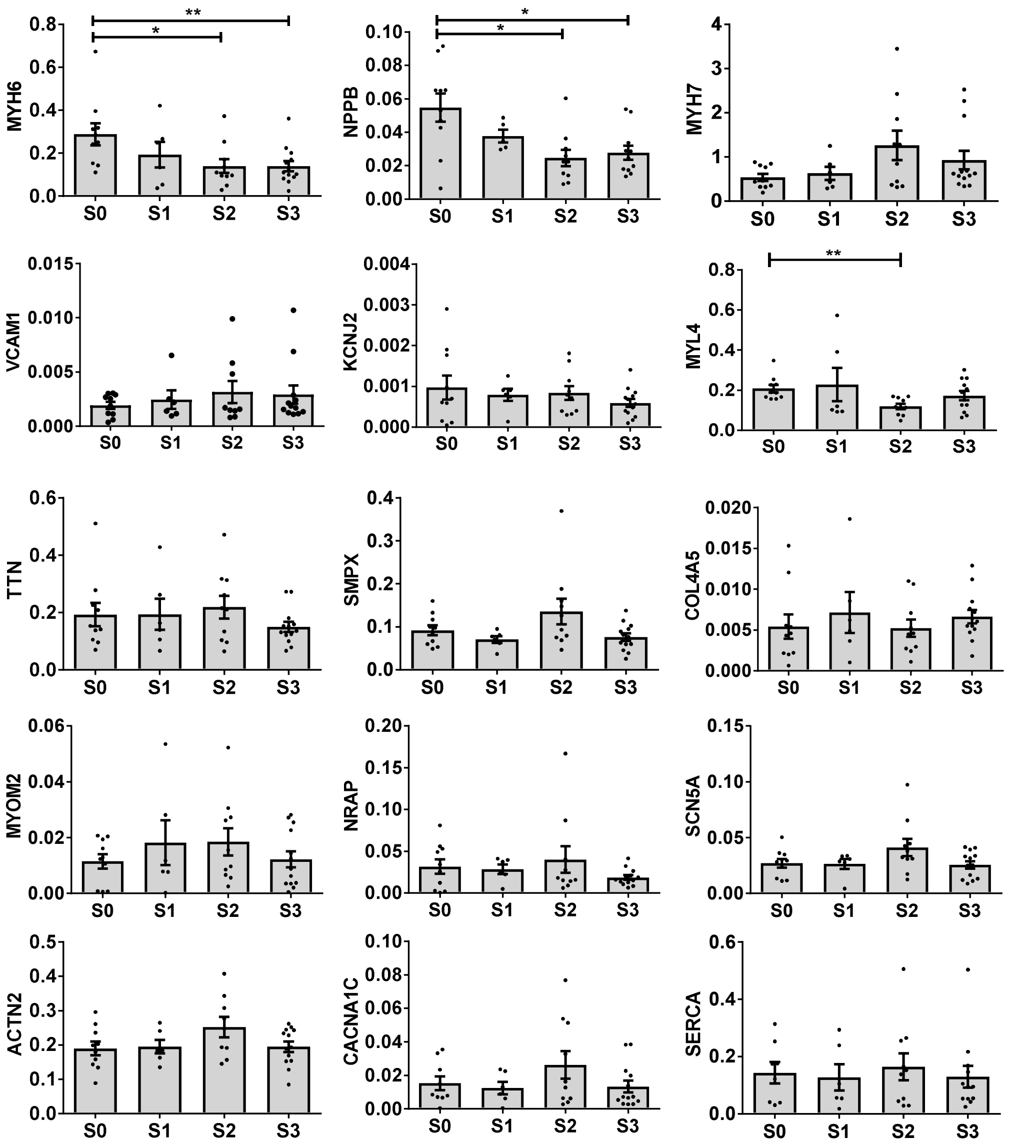

Figure S6. Stretch conditioning modifies expression of myosin isoforms and of brain natriuretic peptide. Bar plots represent messenger RNA abundance of analyzed genes relative to glyceraldehyde 3-phosphate dehydrogenase (GAPDH) in EHTs exposed to different rates of stretch for 21 days, as determined by qPCR. **p < 0.01, unpaired t-test of S0 and S2, S3 groups.
